# Single‐cell transcriptome profiling reveals the key role of ZNF683 in natural killer cell exhaustion in multiple myeloma

**DOI:** 10.1002/ctm2.1065

**Published:** 2022-10-17

**Authors:** Xin Li, Mengping Chen, Yike Wan, Lu Zhong, Xiaofeng Han, Xiaotong Chen, Fei Xiao, Jia Liu, Yiwei Zhang, Di Zhu, Jing Xiang, Junling Liu, Honghui Huang, Jian Hou

**Affiliations:** ^1^ Department of Hematology Ren Ji Hospital Shanghai Jiao Tong University School of Medicine Shanghai China; ^2^ Department of Biochemistry and Molecular Cell Biology Shanghai Jiao Tong University School of Medicine Shanghai China

**Keywords:** exhaustion, multiple myeloma, natural killer cells, SH2D1B, ZNF683

## Abstract

**Backgrounds:**

Decreased cytotoxicity of natural killer (NK) cells has been shown in multiple myeloma (MM). However, the underlying molecular mechanisms remain unclear. Here, by using single‐cell RNA sequencing analysis and in vitro experiments, we aim to uncover and validate molecularly distinctive insights into identifying regulators for NK cell exhaustion and provide potential targets for novel immune therapies in MM.

**Methods:**

Single‐cell RNA sequencing was conducted in the bone marrow and peripheral blood samples from 10 newly diagnosed MM patients and three healthy volunteers. Based on the cluster‐defining differentially expressed genes, we named and estimated functional states of each cluster via bioinformatics analyses. Functional significance of key findings obtained from sequencing analysis was examined in a series of in vitro experiments, including luciferase reporter assay, lentiviral expression vector construction, NK cell transfection, RT‐qPCR, flow cytometry, and cytotoxicity assay.

**Results:**

We classified NK cells into seven distinct clusters and confirmed that a subset of ZNF683^+^ NK cells were enriched in MM patients with ‘exhausted’ transcriptomic profile, featuring as decreased expression of activating receptors and cytolytic molecules, as well as increased expression of inhibitory receptors. Next, we found a significant downregulation of *SH2D1B* gene that encodes EAT‐2, an adaptor protein of activating receptor SLAMF7, in ZNF683^+^ NK cells from MM patients versus healthy volunteers. We further proved that ZNF683 transfection in NK cells significantly downregulated *SH2D1B* expression via directly binding to the promoter of *SH2D1B*, leading to NK cell cytotoxic activity impairment and exhausted phenotypes acquisition. In contrast, ZNF683 knockout in NK cells from MM patients increased cytotoxic activity and reversed NK cell exhaustion.

**Conclusions:**

In summary, our findings uncover an important mechanism of ZNF683^+^ NK cell exhaustion and suggest that transcriptional suppressor ZNF683 as a potential useful therapeutic target in immunotherapy of MM.

## Graphical abstract

Mechanism of ZNF683^+^ NK cell exhaustion in MM microenvironment.

- A subset of ZNF683^+^ NK cells exhibiting exhausted phenotypes were enriched in MM patients.
- ZNF683 transfection significantly downregulates SH2D1B expression via directly binding to its promoter in NK cells, thus attenuating SLAMF7‐mediated activating signals and eventually promoting NK cell exhaustion.
- Knock out ZNF683 expression in NK cells from MM patients reverses their exhaustion phenotypes and promotes cytotoxicity.

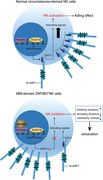

## 1 INTRODUCTION

Multiple myeloma (MM) is a malignant plasma cell disorder, characterized by clonal proliferation of neoplastic plasma cells in bone marrow (BM).^1^ Over the past few years, novel treatment regimens incorporating proteasome inhibitors (PIs), immunomodulatory agents (IMiDs), and monoclonal antibodies (mAbs) have significantly improved clinical outcomes and patient survival.^2^ The immune system is dysregulated along with the progression of MM that allows malignant plasma cells to escape immunosurveillance. Most of the prevalent therapies not only focus on the MM cells but also target the immune system of patients.^3^ In particular, the mAbs such as anti‐SLAMF7 mAb elotuzumab, and anti‐CD38 mAb daratumumab, function in part by facilitating antibody‐dependent cellular cytotoxicity (ADCC) of natural killer (NK) cells,^4^, ^5^ highlighting a pivotal role of NK cells in immune surveillance against MM. Meanwhile, NK cell enhancing strategies are undergoing exploration as adoptive cellular therapies for MM.^6^ Despite efforts in drug development and experimental research to override MM‐induced immunosuppression, patients with MM hardly sustain long‐lasting remissions, and the underlying molecular mechanisms needs further elucidation.

The cytotoxicity of NK cells against virus‐infected and malignant transformed cells is determined by the integration of activating and inhibitory receptors. The most important inhibitory NK cell receptors (iNKRs) are the members of the killer‐cell immunoglobulin‐like receptor (KIR) family and CD94/NKG2A heterodimer.^7^ Additional inhibitory checkpoints also regulate NK cell activation, including CD96, KLRG1, CTLA4, PD‐1, TIGIT, LAG3, TIM‐3, and so forth.^8^ The major activating NK cell receptors (aNKRs) mainly refer to natural cytotoxicity receptors (NCRs), CD16, NKG2D, DNAM‐1, and Signaling Lymphocyte Activation Marker Family member 7 (SLAMF7).^9^, ^10^ Physiologically, iNKRs bind inhibitory ligands on the surface of healthy self‐cells and thereby prevent NK cells from lysing them.^11^ Infected or malignantly transformed cells often lose their surface expression of inhibitory ligands; thus, NK cells are no longer inhibited and are more inclined to activation.^12^ MM cells express a series of activating ligands, including MICA/B, HLA‐E, and PVR, which recognized activating receptors NKG2D, NKG2C, and DNAM‐1 accordingly.^13^ Growing evidence indicates that NK cells in MM microenvironment become dysfunctional by downregulation of aNKRs or upregulation of iNKRs.^14^, ^15^, ^16^ These alterations might potentially impact the response to NK cell‐based therapies. Among receptors expressed on NK cells, SLAMF7 is particularly intriguing in MM for its concurrent and abundant expression on MM cells. SLAMF7 is considered as a self‐adhesion activating receptor on NK cells, and its activating signaling pathway is transduced via adaptor EWS‐FLI1‐activated transcript 2 (EAT‐2).^17^ In the absence of EAT‐2, SLAMF7 mediates inhibitory function in NK cells,^17^ indicating that dysregulation of EAT‐2 might contribute to NK cell dysfunction. However, the relationship between EAT‐2 and NK cell function remains unclear in MM. A better understanding of phenotypes, subpopulation, and function of NK cells in MM microenvironment may provide insights into optimizing treatment strategies, and ultimately lead to more personalized and more effective medicine. Several studies have demonstrated that the development and effector functions of NK cells are regulated by a variety of transcription factors,^18^, ^19^, ^20^ HIF‐1α, SMAD3, and SMAD4 were reported to suppress NK cell‐mediated immunosurveillance in solid tumors.^21^, ^22^, ^23^ Transcription factor ZNF683, a transcriptional suppressor, has been demonstrated to play a key role in regulating NK cell differentiation, while repressing Interferon Gamma (IFN‐γ) production in terminal differentiation stage.^24^ However, the full range of transcriptional modules that regulate NK cell activities have not been fully understood in MM.

In recent years, single‐cell RNA sequencing has become a powerful tool in uncovering intratumor heterogeneity and immune landscape of MM.^25^, ^26^, ^27^ This high‐throughput technology potentially paves way for exploring molecular mechanisms of NK cell dysfunction in MM. In the current study, we performed single‐cell transcriptome analysis of NK cells from MM patients and healthy volunteers to dissect mechanisms for NK cell dysfunction in MM. Based on their transcriptional profiles and effector functions, NK cells were divided into different subsets, then we identified and validated molecularly novel insights into identifying regulators for NK cell exhaustion and provide potential therapeutic targets to improve NK cell response functionality in MM.

## 2 MATERIALS AND METHODS

### 2.1 Primary samples and cell preparation

The study protocols were approved by the Ethics Committee of Ren Ji Hospital, Shanghai Jiao Tong University School of Medicine. Informed consent was obtained from each patient and healthy volunteer in accordance with the Declaration of Helsinki. Sample collection and usage were carried out in strict accordance with institutional guidelines on the experimental use of human tissues.

Samples of BM and PB were obtained from three healthy volunteers and 10 patients with newly diagnosed MM at the Department of Hematology of Ren Ji Hospital. All patients had received Bortezomib, cyclophosphamide and dexamethasone (VCD) regimens. Samples from the included nine patients were obtained upon initial diagnosis and after two cycles of chemotherapy. One patient succumbed due to severe infection, and her post‐treatment samples were not available. As soon as possible after collection, samples were diluted with ice‐cold phosphate‐buffered saline (PBS), carefully layered over Ficoll‐Paque PLUS lymphocyte isolation sterile solution (Cytiva, Sweden), then centrifuged at 670*g* for 20 min at 20°C without braking. Interphase mononuclear cells were carefully transferred to a fresh tube, washed once with PBS, and then used in experiments involving single‐cell RNA sequencing (scRNA‐seq) and flow cytometry.

### 2.2 Sequencing library construction using the 10× genomics platform

An appropriate volume of cell suspension with a concentration of 1,000–1,200 cells/µl was loaded into single cell 3′ chips and mixed with barcoded gel beads on a 10× Chromium Controller (10× Genomics, Pleasanton, CA, USA). RNA from the barcoded cells was subsequently reverse‐transcribed, and sequencing libraries were constructed with reagents from a Chromium Single Cell 3′v3 reagent kit (10× Genomics) according to the manufacturer's instructions and then pooled and sequenced on an Illumina Novaseq6000 according to the manufacturer's instructions.

### 2.3 Raw data generation and processing

Raw reads were demultiplexed and mapped to the reference genome by 10× Genomics Cell Ranger pipeline using default parameters of the Cell Ranger Single‐Cell Software Suite. Single‐cell analyses were performed using Cell Ranger and Seurat unless mentioned specifically. The ‘cellranger count’ was run on FASTQ data from each GEM well individually and then the ‘cellranger aggr’ pipeline was used to aggregate outputs from multiple runs of cellranger count to generate a feature‐barcode matrix. After these runs were normalized to the same sequencing depth, the feature‐barcode matrix was recalculated and analysed. Each gene and each cell barcode were filtered by Cell Ranger, and unique molecule identifiers (UMIs) were counted to construct digital expression matrix. Low‐quality cells were removed with UMI number < 1,000 or with over 10% mitochondrial‐derived UMI counts. Secondary filtration, variable gene selection, dimensionality reduction, clustering, and visualization were performed using Seurat Second QC, where a gene with expression in more than three cells was considered expressed, and cells with fewer than 200 expressed genes were excluded. Single cells with over 6,000 genes detected were also filtered out in order to eliminate potential doublets. After preprocessing, DoubletFinder43 (v2.0) was used to identify putative doublets in each dataset, individually. BCmvn optimization was used for PK parameterization. Estimated doublet rates were computed by fitting the total number of cells after quality filtering to a linear regression of the expected doublet rates published in the 10× Chromium handbook. Estimated homotypic doublet rates were also accounted for using the modelHomotypic function. The default PN value (0.25) was used. Putative doublets were then removed from each individual dataset. Finally, 2,41,440 single cells remained, and they were applied in downstream analyses. QC plots after filtering were displayed in Figure S1A.

### 2.4 Typing of immune cell types and clustering of NK cells

The feature‐barcode matrices produced by cellranger count were reanalysed by cellranger and the dimensionality reduction, clustering, and gene expression algorithms were rerun using the cellranger default parameter settings. After quality control, the UMI count matrix was log normalized. Since the sample from 13 patients were processed and sequenced in batches, patient number was used to remove potential batch effect. We merged the datasets and performed batch‐correction with fastMNN (PMID: 29608177). We then used Seurat to process the integrated data. The UMI count matrices were log normalized using the Seurat package (v3.2.0).^28^ To reduce the dimensionality of the scRNA‐Seq dataset, we performed principal component analysis (PCA) on the integrated data matrices based on selected highly variable genes to build a graph which was segmented with a resolution of 0.6. Cells were clustered using unsupervised graph‐based clustering on the informative PCA space.^29^ Data annotated with corresponding clusters were visualized by uniform manifold approximation and projection (UMAP). Cells were annotated as different major immune cell types based on their mean gene expression of known marker genes, including *CD3D*, *CD14*, *NKG7*, *LCN2*, *CD138*, *CD20*, *MPO*, *HBB*, *CD1C*, *CD10*, *CD34*, *CD123*, and *PPBP*.

NK cells were clustered into subtypes based on contrasting marker gene expression profiles, as determined using the ‘FindAllMarkers’ function in Seurat with the default two‐sided, nonparametric Wilcoxon rank sum test. Genes were considered as differentially expressed genes (DEGs) between clusters if the Bonferroni‐adjusted *P* < .05 and if the mean fold change in expression (natural log‐transformed) was at least .25.

### 2.5 Pathway enrichment analysis

Global DEGs of cell subgroups were recognized based on filtered gene expression matrix by Seurat. Differential expression analysis was carried out using the edgeR package to obtain zone‐specific marker genes. To gain functional and mechanistic insights of cell clusters, we performed KEGG Pathway enrichment analyses using the clusterProfiler R package (v3.14.3), in which gene length bias was corrected, to identify biological pathways that were enriched in marker genes. Corrected *P* < .05 were considered significantly enriched.

### 2.6 Pseudotime and trajectory analysis

The pseudotime and trajectory analysis was performed using Monocle 2 software (Monocle 2.14.0) with default settings.^30^ NK cells in clusters 3, 5, and 6 were analysed for deducing pseudotime trajectory. The group‐specific marker genes were selected using the ‘detectGenes’ function. Next, we pseudo‐temporally ordered cells using the ‘reduceDimension’ and ‘orderCells’ functions. The expression dynamics along the trajectories were visualized using the BEAM function.

### 2.7 NK cell isolation and expansion

NK cells ready for transfection, as well as following flow cytometry and RT‐qPCR were purified from healthy volunteers‐derived PBMCs (*n* = 3) and MM patients‐derived PBMC (*n* = 3), according to the manufacture's instruction of the Human NK cell Isolation Kit (Miltenyi Biotec, Cologne, Germany). Then, the cells were fluorescently stained with PE anti‐human CD56 antibody (Biolegend, California, USA) and FITC anti‐human CD3 antibody (Biolegend) and analysed by flow cytometry to guarantee a purity of more than 95% CD3‐CD56 + NK cells. Expansion procedures have been described by Wagner et al.^31^ Briefly, K562‐based artificial antigen‐presenting cells expressing membrane‐bound interleukin (IL)‐21 (K562‐mb21‐41BBL feeder cells) were used to expand NK cells. They were irradiated at 100 Gy and added at a feeder cell: PB‐NK ratio of 10:1. NK cells (1 × 10^6^) were cultured in SCGM medium (CellGenix, Portsmouth, NH) supplemented with 10% fetal bovine serum (FBS, Gibco, Grand Island, USA), 400 U/ml recombinant human IL‐2 (R&D Systems, Minneapolis, MN), and K562 cells at 1 × 10^7^. During the 7‐day culture, IL‐2 and freshly prepared K562 cells were replenished every 2 days.^32^

### 2.8 Construction of lentiviral expression vectors and NK cell transfection

The lentiviral vector PGMLV‐CMV‐MCS‐EF1‐ZsGreen1‐T2A‐Puro (Genomeditech) containing *ZNF683* cDNA were used for gain‐of‐function experiments. The lentiviral construct PGMLV‐HU6‐MCS‐CMV‐ZsGreen1‐PGK‐Puro encoding short‐hairpin RNA (shRNA) targeting *SH2D1B* and *ZNF683*, or the corresponding scrambled sequences as negative controls (Table S1) were obtained. Constructs were co‐transfected with packaging plasmids (TIANGEN) into HEK293 cells to obtain recombinant lentivirus, which was added to cell cultures for 24 h in the presence of 8 µg/ml polybrene (Genomeditech). To enhance the efficacies of NK cell transfection, we employed a novel spinfection protocol.^33^ Briefly, NK cells were activated for 5–7 days as described above, then mixed with lentivirus at a multiplicity of infection (MOI) of 10 and 5 µg/ml polybrene, they were centrifuged at 1000 × g for 1 h at ordinary temperature subsequently. After 5 days, transfection efficacies were assessed by RT‐qPCR before each experiment.

### 2.9 Quantitative reverse transcription‐polymerase chain reaction (RT‐qPCR)

Total cellular RNA was isolated from 1 × 10^5^ lentiviruses‐infected cells using a RNeasy Kit (Qiagen, Germany) as instructed by the manufacturer, reverse‐transcribed into complementary DNA (cDNA) using HiScript III All‐in‐one RT SuperMix (Vazyme, China), and subjected to quantitative real‐time RT‐PCR with *GAPDH* as an endogenous control. cDNA was quantified using ChamQ SYBR Color qPCR Master Mix (Vazyme). The sequences of the PCR primers were listed in Table S2. PCR was performed at 94°C for 4 min, followed by 40 cycles of 94°C for 1 min, 56°C for 1 min, and 72°C for 1 min. Expression of *ZNF683* and *SH2D1B* was normalized to that of *GAPDH* using the 2^ΔΔCT^ method.

### 2.10 Online database analysis

To validate the regulatory relationship between transcription factor ZNF683 and its target genes, the ZNF683‐binding motif sequence predicted by the JASPAR database (http://jaspar.genereg.net/) was mapped to predicted promoter regions (TSS ± 2 kb) of target gene.

### 2.11 Luciferase reporter assays

The luciferase reporter assay was performed to examine the relationship between transcription factor and its predicted target genes.^34^ Briefly, HEK293 cells were co‐transfected with the constructs pcDNA3.1(+)‐H_ZNF683 and PGL3‐basic‐H_*SH2D1B* promoter were cultured for 48 h under low‐light conditions.^34^ Then, cells were fully lysed by cell lysis buffer (Genomeditech); the firefly luciferase (LUC)‐Renilla luciferase (REN) activity ratio was determined for the luciferase reporter system (Tecan microplate reader). The LUC–REN ratio indicated transcriptional activity.

### 2.12 Flow cytometry

NK cells were isolated as described above and incubated for 20 min at 4°C with antibodies against CD158a (Beckman Coulter, CA, USA), CD158b (Beckman Coulter), CD56 (Beckman Coulter), or CD16 (Beckman Coulter) or antibodies against LAG3 (Biolegend), TIGIT (Biolegend), CTLA4 (Biolegend), CD57 (Biolegend), NKG2A (Biolegend), CD107A (Biolegend), Nkp46 (Biolegend), or NKG2D (Biolegend). After membrane staining, the cells were fixed with Fixative Reagent (Beckman Coulter) at room temperature for 15 min, then permeabilized with 300 µl permeabilizing reagent (Beckman Coulter) to detect intracellular protein expression. Permeabilized cells were incubated for 15 min at room temperature with antibodies against Granzyme B (Biolegend), perforin (Beckman Coulter), or IFN‐γ (Beckman Coulter). Flow cytometry was conducted on a DxFlex system (Beckman Coulter), and data were analysed using FlowJo software (v10.5.3).

### 2.13 Cytotoxicity assays

Cytotoxicity were tested using luciferase‐labelled target cells (Luc‐Raji) as described by Melaiu et al.^35^ Luc‐Raji cells were suspended in RPMI‐1640 medium supplemented with 10% FBS in 96‐well, flat‐bottomed white view plates (Corning). Expanded NK cells suspended in RPMI 1640 with 10% FBS and 400 IU/ml IL‐2 were added at varying effector to target cell ratios as indicated in results section. The plates were incubated for 4 h at 37°C with 5% CO_2_. At the end of the culture, cell mixture was incubated with equal volume of D‐luciferin bioluminescent substrate (Perkin‐Elmer, Waltham, USA) for 10 min, then measured using a microplate reader (SpectraMax iD3). Viability was calculated by comparing relative luminescent signal from control wells on each plate. All experiments were done in triplicates.

### 2.14 Statistics

All statistical analyses were performed using GraphPad Prism (version 8.3.0), or R (version 3.6.0). Data are shown as mean values ± standard deviation (SD). Intergroup differences were assessed for significance using the unpaired Student's *t* test. The correlation between the read count values of genes was evaluated using Spearman correlation coefficient. Nonparametric Mann‐Whitney tests were applied to compare cluster distribution between MM patients and healthy volunteers. Considering the relatively unbalanced sample size between MM patients and healthy volunteers, we normalized the data for numbers of cells in each group by random subsampling to better dissect the NK cell compartment. Differences associated with *P* < .05 were considered significant; ns, not significant; *P < .05; **P < .01; ***P < .001; ****P < .0001.

## 3 RESULTS

### 3.1 Single‐cell transcriptome analysis revealed that a distinct ZNF683^+^ NK subset is enriched in MM

To better understand the discrepancies of immune microenvironment across MM patients and healthy individuals, we performed single‐cell RNA sequencing on mononuclear cells from BM and PB of 10 newly diagnosed MM patients and three healthy volunteers (Table 1). The median age of MM patients and healthy volunteers were 55 and 53 years old, respectively, and the resuming ratios between F and M were almost equal. Among MM patients selected, 50% of the patients had high‐risk cytogenetic abnormalities, and most patients were at intermediate/high stages (≥ Stage II) of disease, assessed by ISS (70%) and R‐ISS (80%) stage systems.^36^

**TABLE 1:** Clinical information of enrolled MM patients and healthy control

	MM patients (*n* = 10)	Healthy volunteers (*n* = 3)
**Age**		
Median, year	55 (41–64)	53 (50–56)
**Sex**		
Female	5 (50%)	1 (33.33%)
Male	5 (50%)	2 (66.67%)
**Type of disease**		
IgG, κ	7 (70%)	N/A
IgG, λ	1 (10%)	N/A
IgA, λ	1 (10%)	N/A
Light chain (κ)	1 (10%)	N/A
**Chromosomal abnormality – no. (%)**		
High risk overall	6(60%)	N/A
del(17p)/p53	1(10%)	N/A
t(4;14)	5(50%)	N/A
gain (1q)	5(50%)	N/A
**International Staging System (ISS) stage**		
I	3 (30%)	N/A
II	6 (60%)	N/A
III	1 (10%)	N/A
**R‐ISS stage**		
I	2(20%)	N/A
II	8(80%)	N/A
III	0	N/A

Abbreviations: MM; NEG, negative; N/A, data not available; NDMM, newly‐diagnosed.

**FIGURE 1:**
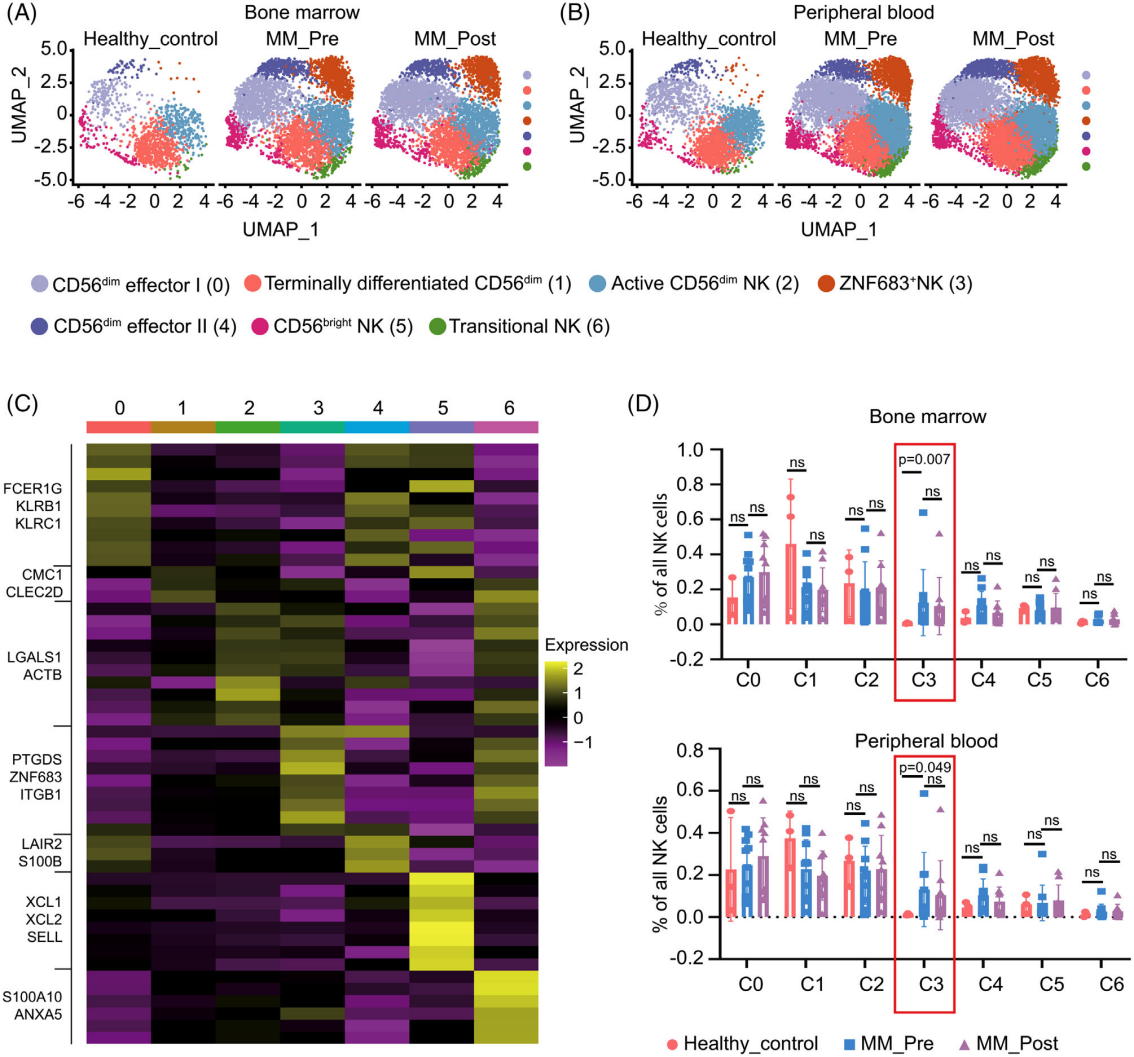
Single‐cell transcriptome profiles of NK cells from MM patients and healthy volunteers‐derived BM/PB samples. (A and B) Uniform Manifold Approximation and Projection (UMAP) visualization of 30,008 single NK cells from healthy volunteers (Healthy_control, *n* = 3), MM patients before treatment (MM_Pre, *n* = 10), and after treatment (MM_Post, *n* = 9). NK cells from both BM (A) and PB (B) are colour‐coded and divided into seven clusters according to the expression levels of marker genes. (C) Marker gene analyses identify heterogeneous clusters as CD56^dim^ effector I NK, terminally differentiated CD56^dim^ NK, active CD56^dim^ NK, ZNF683^+^ NK, CD56^dim^ effector II NK, CD56^bright^ NK, and transitional NK. Expression of the top marker genes for each cluster is depicted as heatmap. (D) The percentages of each NK cluster in total NK cells of BM samples (upper panel) and PB samples (lower panel)

A total of 2,41,440 single cells were divided into 13 clusters (Figure S1B,C). These clusters mainly included T cells, monocytes/macrophages, plasma cells, NK cells, and a small proportion of B cells. Through unsupervised clustering, 30,008 NK cells were further divided into seven subgroups (0–6) and visualized using UMAP plots (Figure 1A,B).

As shown in Figure S2A, cluster 0 to cluster 4 showed low expression of *NCAM1* (CD56); thus, they were recognized as CD56^dim^ NK cells. Based on the cluster‐defining differentially expressed genes, we termed the five CD56^dim^ NK clusters as ‘CD56^dim^ effector I NK’, ‘terminally differentiated NK’, ‘active CD56^dim^ NK’, ‘ZNF683^+^ NK’, and ‘CD56^dim^ effector II NK’ (Figure S2B–E).^24^, ^37^, ^38^, ^39^, ^40^ Cluster 5 was identified as ‘CD56^bright^ NK’ cells, for their high expression of *NCAM1*(CD56), together with *IL7R* (IL7RA), *SELL* (CD62L), and *GZMK* (granzyme K, Figure S2A,F).^39^, ^41^ Finally, cluster 6 was transcriptionally similar to the ‘CD56^bright^ NK’ cluster. Remarkably, this cluster also overexpressed genes associated with CD56^dim^ NK cells, such as CD16A (*FCGR3A*) and downregulated hall marker genes of CD56^bright^ NK cell, including *KLRC1*, *SELL*, and *GZMK* (Figure S2E,F), referring to previously described ‘transitional NK’.^38^

To investigate the relative contribution of different NK clusters to the microenvironmental repertoire, we next compared compositional alterations of NK cells between MM patients and healthy volunteers. Considering plasma cells are specifically abundant in MM‐derived samples, we excluded them from total mononuclear cell counts when calculating NK cell proportion. As shown in Figure S3A,B, the proportion of NK cells in plasma cell‐depleted mononuclear cells showed no statistical significance between MM patients and healthy volunteers in both BM and PB samples. We further compared the proportion of each NK cluster in total NK cells between BM and PB in healthy volunteers, MM patients before and after treatment, and found that there was no statistical significance in the proportion of NK cell subsets in BM and PB (Figure S3C–E). However, we observed a significant enrichment in ZNF683^+^ NK cells (cluster 3) from both MM patients‐derived BM and PB samples (Figure 1A,B). The percentages of ZNF683^+^ NK cells among total NK cells were significantly higher in MM patients than healthy volunteers in BM (a mean of 16.5% over .7%, *P* = .007) and PB (16.1% vs. 1.2%, *P* = .049), but there was no significant difference in percentages before and after treatment in both BM and PB (Figure 1D) in MM patients. The number of other NK cell subsets has not significantly changed in MM patients and healthy volunteers, nor did they alter before and after treatment. We further explored C3 enrichment in MM patients with high‐risk FISH features^42^ and malignant plasma cell BM infiltration. Ten MM patients were divided into high‐risk (*n* = 6) and standard‐risk (*n* = 4) two groups, according to FISH features. The results indeed revealed that significantly higher ratio of high C3 enrichment (4/6 vs. 1/4) in high‐risk group versus standard‐risk group (Fisher's Exact Test, *P* = .008). Moreover, we found that high C3 enrichment is associated with high‐risk R‐ISS stage (Fisher's Exact Test, *P* = .022). However, there seems to be no obvious correlation between C3 enrichment and malignant plasma cell BM infiltration. Remarkably, there are two points (one MM pre and one post) from the same patient that display a higher C3 distribution both in BM and in PB. This patient had both t(4;14) and TP53 mutations, and highest C3 distribution among the 10 MM patients.

To comprehensively investigate the crosstalk between C3 and other cellular components in the immune microenvironment, we investigated the intercellular communication among ZNF683^+^ NK cells, exhausted T cells, and plasma cells of MM patients before and after treatment by Cellcall (v0.0.0.9000). The results of these analyses showed diminished intercellular communication between ZNF683^+^ NK cells and exhausted CD8^+^T cells after treatment (vs. prior to treatment), indicating that communications between inhibitory immune cells were partly reversed along treatment (Figure S3F,G).

### 3.2 ZNF683^+^ NK cells exhibit decreased cytotoxicity‐related markers in myeloma

**FIGURE 2:**
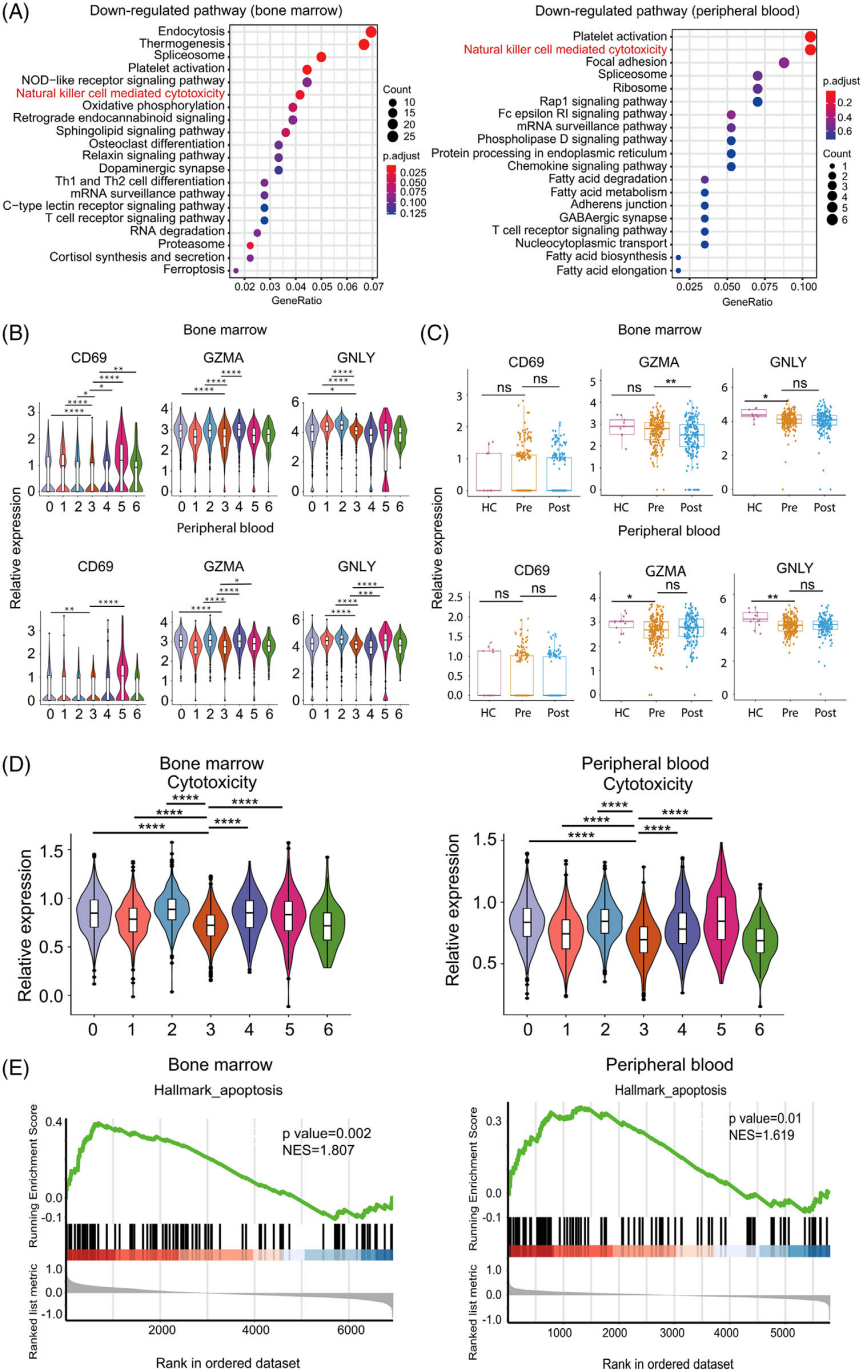
Expression of cytotoxicity‐related markers in ZNF683^+^ NK cells. (A) KEGG analyses exhibit down‐regulated pathways in BM and PB MM‐derived ZNF683^+^ NK cells in comparison with healthy volunteers. (B) Violin plot show expression level of traditional cytolytic molecules *CD69*, *GNLY* (encoding for granulysin), and *GZMA* (encoding for granzyme A) in each NK cell cluster from BM and PB. *P < .05; **P < .01;***P < .001; ****P < .0001, by Wilcoxon rank‐sum test. (C) Box plots compare *CD69*, *GZMA*, and *GNLY* expression in ZNF683^+^ NK cells between MM patients (Pre) and healthy volunteers (HC). *P < .05; **P < .01, by Wilcoxon rank‐sum test. (D) Cytotoxicity score in all 7 NK cell clusters. (E) GSEA plots reveal pathways enriched in MM patients‐derived ZNF683^+^ NK cells compared with healthy volunteers. NES, normalized enrichment score, by Kolmogorov‐Smirnov test

To compare functional discrepancies of ZNF683^+^ NK cells between MM patients and healthy individuals, we conducted KEGG pathway enrichment analyses and found that the NK cell‐mediated cytotoxicity pathway was significantly downregulated in both BM and PB ZNF683^+^ NK cells from MM patients than these from healthy volunteers‐derived ZNF683^+^ NK cells (Figure 2A). To further explore cytotoxicity‐related molecule expression difference between MM patients and healthy volunteers, we selected 18 genes involved in NK cell‐mediated cytotoxicity KEGG pathway and found that three cytotoxic genes including *SH2D1B* (FDR correction, *P* < .0001), *SYK* (FDR correction, *P* = .018), and *FCER1G* (FDR correction, *P* < .0001) were significantly downregulated in BM‐derived ZNF683^+^ NK cells of patients relative to healthy volunteers, together with cytotoxic gene *SH2D1B*(FDR correction, *P* < .0001) were significantly downregulated in PB‐derived ZNF683^+^ NK cells of patients to healthy volunteers (Figure S4A,B). We further found that ZNF683^+^ NK cells exhibited lower expression of the traditional cytolytic molecules *CD69*, *GNLY* (encoding for granulysin), and *GZMA* (encoding for granzyme A) than most of other NK cell clusters did (Figure 2B). Moreover, the expression of *GZMA* and *GNLY* were significantly lower in patient‐derived ZNF683^+^ NK cells than these from controls based on analysis of BM and/or PB (Figure 2C). We further explored the expression of other cytokines including Tumor Necrosis Factor (TNF, CCL3, CCL4, XCL1, and IL10 in ZNF683‐expressing NK cells. The expressions of these cytokines in ZNF683^+^ NK cells were significantly lower than in most of other NK cell clusters in both BM and PB samples (Figure S4C). To better examined the phenotypes of ZNF683^+^ NK cells, we scored all NK cell clusters by known cytotoxicity signatures and noticed that ZNF683^+^ NK cells had lower cytotoxic score than other clusters as well (Figure 2D), providing more evidence for ZNF683^+^ NK cell potential dysfunction in MM patients. GSEA (Gene Set Enrichment Analysis) further showed that apoptosis‐associated genes in ZNF683^+^ NK cells were highly enriched in patients relative to controls in both BM and PB (Figure 2E). Collectively, these data suggest that ZNF683^+^ NK cells become dysfunctional in MM microenvironment.

### 3.3 ZNF683^+^ NK cells exhibit exhaustion phenotypes in MM

**FIGURE 3:**
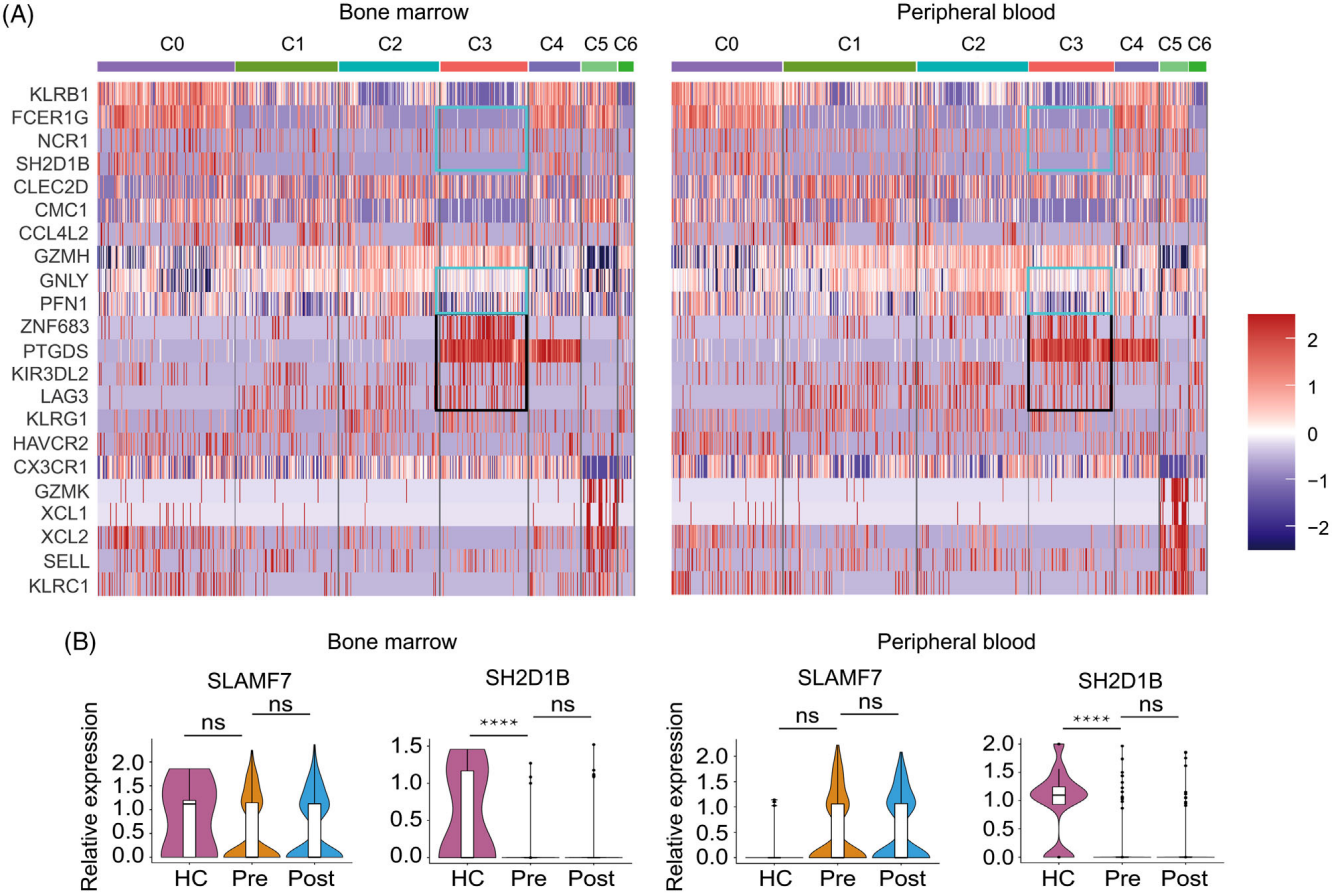
Phenotypes of ZNF683^+^ NK cells in MM patients. (A) Representative DEGs in each cluster of MM patients‐derived NK cells. (B) Violin plots depicts expression of activating receptor *SLAMF7* and its adaptor gene *SH2D1B* in ZNF683^+^ NK cells in BM/PB from healthy volunteers (HC) and MM patients (Pre). ****P < .0001, by Wilcoxon rank‐sum test. *Abbreviation*: NS, non‐significant

To clarify the phenotype characteristics of the dysfunctional ZNF683^+^ NK cells in MM patients, in addition to cytotoxicity markers mentioned above, we compared activating/inhibitory receptor expression in ZNF683^+^ NK cells and other NK cell clusters in MM patients. As shown in Figure 3A, ZNF683^+^ NK cells from MM patients exhibited higher expression of NK cell inhibitory markers *LAG3* (*P* < .0001) and *KIR3DL2* (*P* < .0001) than other NK cell clusters. However, we found that the expression of activating receptor *NCR1*, together with cytotoxicity‐associated markers *PFN1*, *GNLY*, and *FCER1G*, were significantly downregulated in ZNF683^+^ NK cells than other clusters. Furthermore, we compared expression of aNKRs and iNKRs between MM patients and healthy volunteers. Similarly, most aNKRs, including *NCR1*, *NCR3*, *CD244* (2B4), and *CD226* (DNAM‐1) showed a tendency of expression decrease in MM‐derived ZNF683^+^ NK cells. Among them, *NCR1* was significantly downregulated in patients PB samples than healthy volunteers (*P* = .026, Figure S5A). Meanwhile, we examined the expression of iNKRs on ZNF683^+^ NK cells. The results demonstrated that most iNKRs were upregulated in MM patients than healthy individuals with a spectrum of inhibitory receptors including *LAG3*, *KLRG1*, and *KIR* remarkably upregulated in ZNF683^+^ NK cells (Figure S5B). For most of these iNKRs, their upregulation in MM patients was greater in BM than in PB (Figure S5C), indicating that the extent of NK cell exhaustion was more profound in BM than these in PB.

It is worth noting that among aNKRs expressed on ZNF683^+^ NK cells, *SLAMF7* expression did not differ significantly between patients and healthy individuals. However, expression of SLAMF7 adaptor EAT‐2 gene (*SH2D1B*) was almost absent in ZNF683^+^ NK cells from MM patients, significantly lower than that in healthy volunteers (*P* < .0001 in BM, and *P* < .0001 in PB, Figure 3B). Therefore, *SLAMF7* might fail to exhibit activating function in ZNF683^+^ NK cells due to *SH2D1B* absence.

Exhaustion, anergy, and senescence are three different forms of NK dysfunction. Except for exhaustion markers described above, we further monitored expression of anergy and senescence markers in ZNF683^+^ NK cells. NK cells anergy results from attenuation of 4‐1BB activation, which tend to be the case when NK cells are infiltrating in MHC class I‐deficient tumours. NK cells in anergic states are featured as decreased expression of ADCC‐mediating marker CD16 and degranulation marker CD107a.^43^, ^44^ In our research, there was no significant difference in expression of the anergy marker gene *FCGR3A* (CD16) and *LAMP1* (CD107A) between patients and controls (Figure S6A). Similarly, no significant difference was observed in expression of the senescence marker *B3GAT1* (CD57) and *KLRC1* (NKG2A) between patients and controls (Figure S6A).^43^ We further examined CD16, as well as CD107a, NKG2A, and CD57 expression via flow cytometry. As shown in Figure S6B, there was no significant effect on membrane expression of anergy and senescence markers in ZNF683 overexpression NK cells.

**FIGURE 4:**
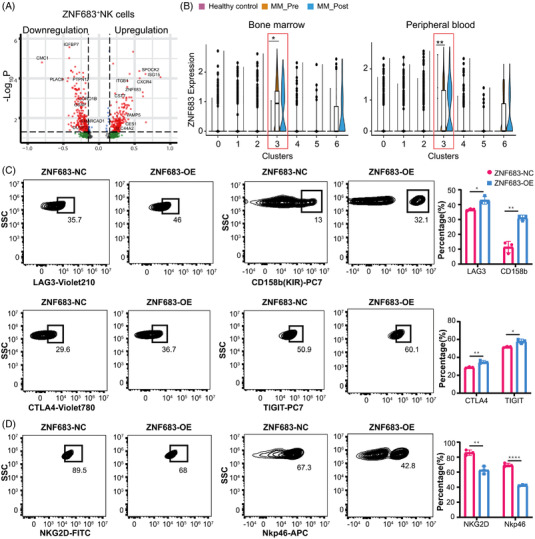
The effect of ZNF683 transfection on NK cell phenotype alterations. (A) Volcano plot depicts the upregulated (right panel) and downregulated genes (left panel) in MM‐derived ZNF683^+^ NK cells compared with these in healthy volunteers. (B) Violin plots show *ZNF683* expression among NK subsets in BM/PB from healthy volunteers and MM patients. *P < .05, **P < .01, by Wilcoxon rank‐sum test. (C) NK cells were isolated from PB of healthy volunteers (*n* = 3), then they were transfected with ZNF683 overexpressing vectors (ZNF683‐OE) or empty vectors (ZNF683‐NC). Flow cytometry results demonstrate the effect of ZNF683 transfection on expression of inhibitory receptors LAG3, CTLA4, TIGIT, CD158b. (D) Flow cytometry result demonstrates the effect of ZNF683 transfection on expression of activating receptor NKG2D and NKp46. For C and D, *P < .05, **P < .01, ****P < .0001, by two‐tailed Student's *t* test

Unlike similar expression of anergy and senescence markers between MM patients and healthy volunteers, exhaustion markers were significantly upregulated in MM‐derived ZNF683^+^ NK cells as described above, indicating that dysfunctional ZNF683^+^ NK cells in MM might exhibit exhausted phenotypes. Remarkably, *ZNF683* expression was observed to be much higher in MM patients‐derived ZNF683^+^ NK cells than these from healthy volunteers, in both BM and PB samples (Figure 4A,B), indicating that ZNF683 might play a pivotal role in the process of NK cell exhaustion.

### 3.4 Transfection of transcription factor ZNF683 promotes exhaustion and reduces cytotoxicity of NK cells

As ZNF683^+^ NK cells performed exhausted phenotypes and behaved dysfunction, we further study the role of ZNF683 overexpression in NK cell exhaustion and identify relevant molecules and pathways. We firstly evaluated the impact of ZNF683 transfection of NK cells isolated from healthy volunteers (*n* = 3) on the expression of exhaustion‐related markers. Compared with NK cells with empty vector (EV) transfection, ZNF683 transfection significantly induced upregulation of inhibitory receptors LAG3(*P =* .0167), CTLA4 (*P* = .0064), TIGIT (*P* = .0134), and CD158b (KIR, *P* = .0013) (Figure 4C, Figure S7A), as well as downregulation of activation receptor NKG2D (*P* = .0030) and NKp46 (NCR, *P*<.0001, Figures 4D and S7B). Our results indicate that transfection of ZNF683 induces exhaustion‐related phenotypes in NK cells.

**FIGURE 5:**
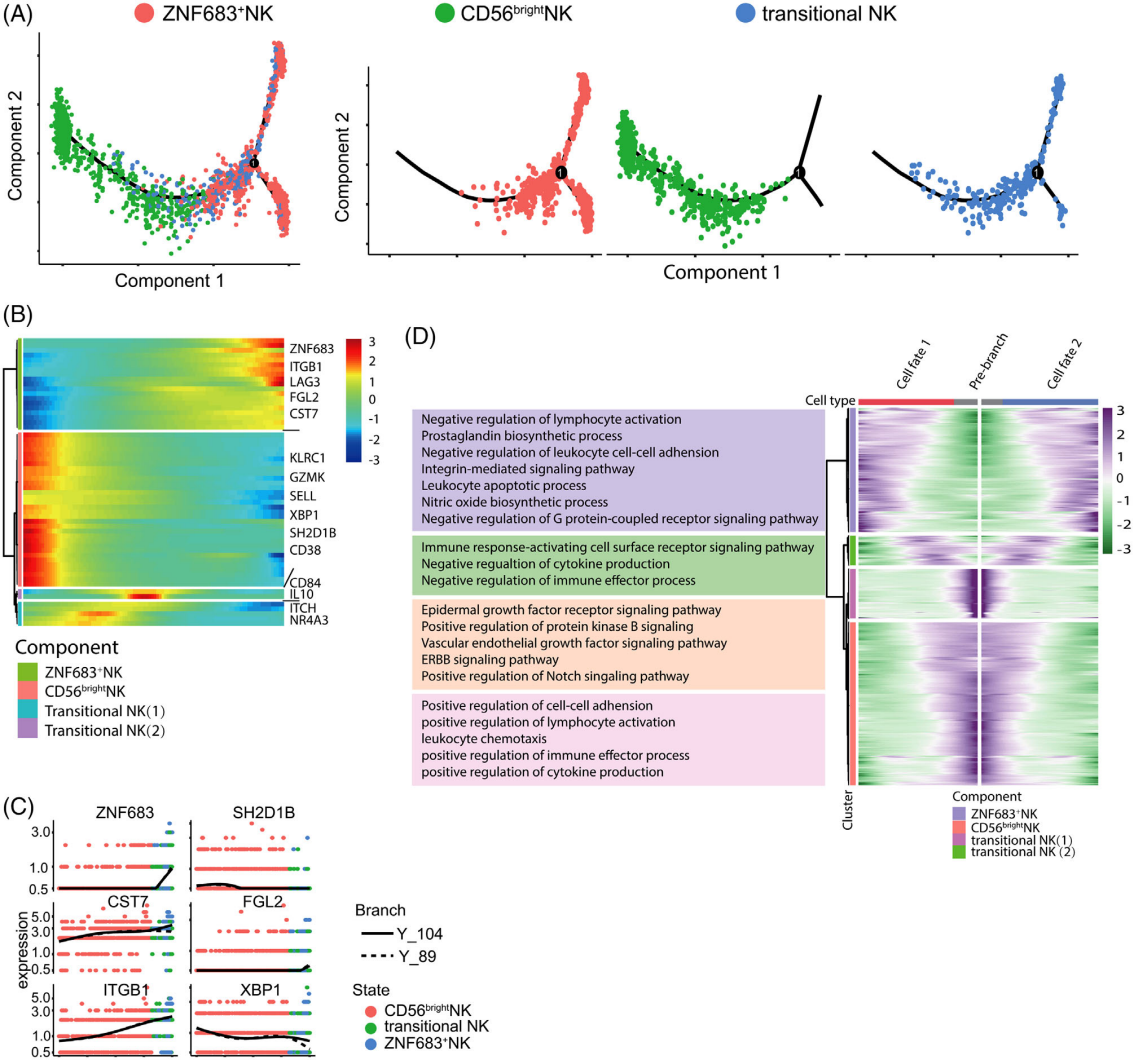
Gene expression dynamics along the pseudotime of NK cell exhaustion. (A) pseudotime trajectory analysis of ZNF683^+^NK cells, transitional NK cells and CD56^bright^ NK cells with high variable genes. Each dot represents one single cell, colour‐coded according to its cluster label. The integrated diffusion maps of clusters of NK cells reveal a developmental trajectory with conserved ‘pseudo‐temporal’ dynamics. (B) Heatmap depicting expression of state‐specific markers obtained by monocle. (C) Gene expression variation in ZNF683^+^NK cells relative to immature NK cells were shown. (D) Heatmap show GO analysis results of state‐specific signalling pathways along the pseudo‐time

To investigate how NK cells develop exhausted phenotypes in MM, we performed the trajectory analysis using ‘CD56^bright^ NK’ (cluster 5), ‘transitional NK’ cells (cluster 6), and ‘ZNF683^+^ NK’ (cluster 3). As ‘CD56^bright^ NK’ subset represents immature NK cells, ‘transitional NK’ subset refers to NK cells in the middle of development, the integrated diffusion maps of NK clusters revealed a developmental trajectory with ‘pseudo‐temporal’ dynamics. ZNF683^+^ NK cells were proved to be at the terminal stage of the developmental trajectory (Figure 5A). To test whether the expression dynamics of ZNF683 would coincide with the transition from immature CD56^bright^ NK to exhausted ZNF683^+^ NK cells when ZNF683 promotes NK cell exhaustion, we further analysed the expression patterns of all detected genes along the trajectory of NK cell exhaustion and identified 3238 genes with dynamic expression changes. ZNF683 expression was confirmed to be gradually upregulated along the pseudotime of NK cell exhaustion. Besides, we observed the expression of proexhaustion genes, such as *LAG3*, *CST7*, and *ITGB1* were upregulated, while activation‐related genes like *SH2D1B* and *XBP1* were downregulated in line with NK cell exhaustion, these dynamic alterations partly explained why ZNF683^+^ NK cells and transitional NK cells (cluster 6) had similar expression patterns of several genes, and we consider transitional NK cells might be a pre‐exhaustion subset (Figure 5B,C). Functional enrichment analysis further showed that enriched genes in ZNF683^+^ NK cells were associated with functions such as negative regulation of leukocyte activation, negative regulation of cell adhesion, while enriched genes in immature CD56^bright^ NK cells positively regulated cell adhesion and activation (Figure 5D). Taken together, our results suggest that NK cell exhaustion in MM patients are resulted from ZNF683 overexpression along with the developmental trajectory of NK cells.

**FIGURE 6:**
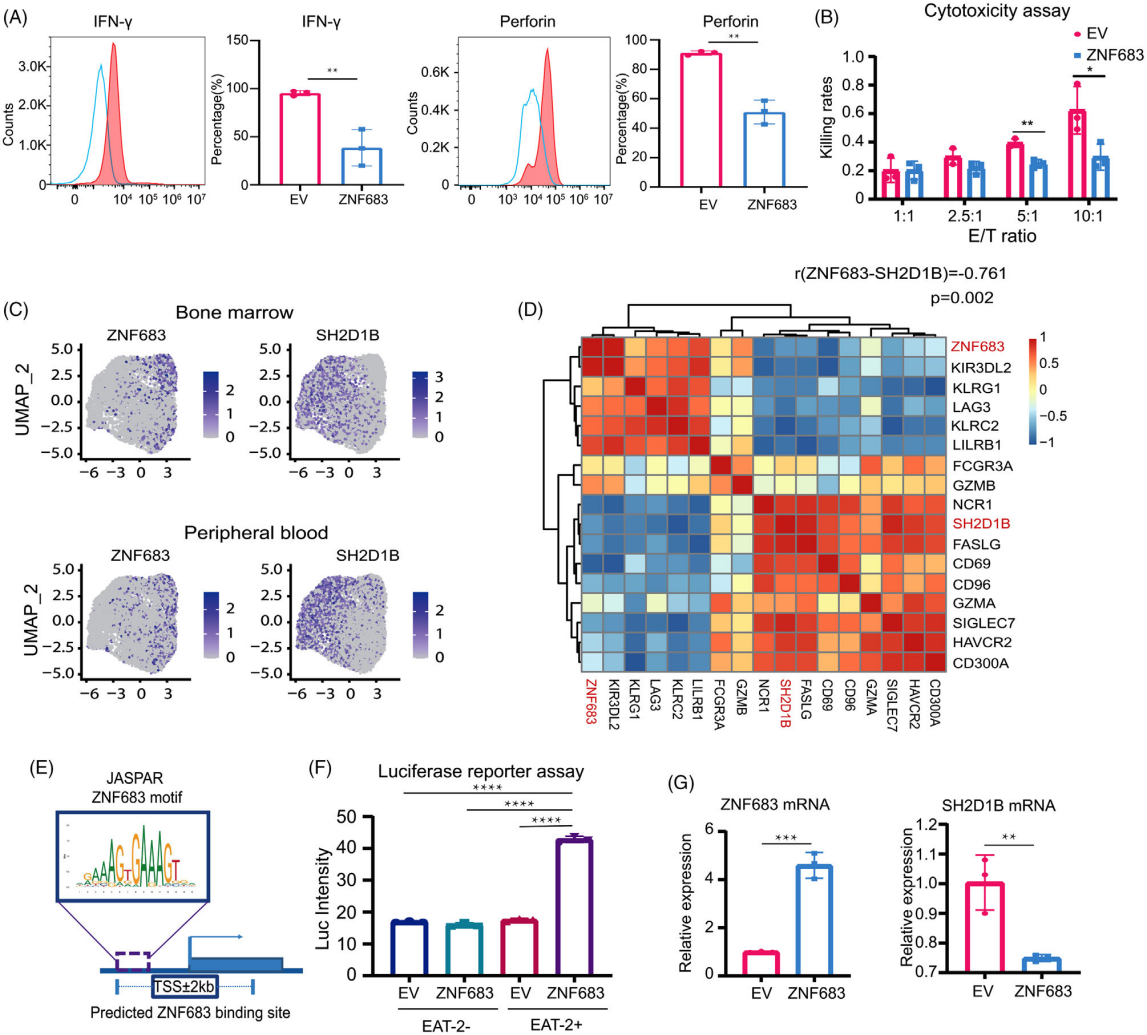
The effect of ZNF683 transfection on NK cell cytotoxicity and regulatory relationship between ZNF683 and *SH2D1B*. (A) Flow cytometry results demonstrate the effect of ZNF683 transfection on IFN‐γ and perforin expression. (B) Cytotoxicity assay illustrated the effect of ZNF683 transfection on NK cell cytotoxicity (*n* = 3 per group). (C) Feature plot revealed *ZNF683* and *SH2D1B* expression in diverse NK cell clusters. (D) Correlation analysis reveals the relationship between *ZNF683* and *SH2D1B*. The correlation between genes was evaluated using Spearman correlation coefficient (r= ‐.745, *P*= .002) and Pearson's correlation coefficient (r= ‐.761, *P*= .002). (E) Predicted ZNF683 binding site sequence on *SH2D1B* from database JASPAR. (F) Luciferase reporter assay depicts regulatory relationship between ZNF683 and *SH2D1B*. (G) NK cells isolated from PB of healthy volunteers (*n* = 3) were transfected with ZNF683 overexpressing vectors or EV. RT‐qPCR revealed the effect of ZNF683 transfection on *SH2D1B* expression in NK cells. *P < .05, **P < .01, ***P < .001, ****P < .0001, by two‐tailed Student's t test

Our finding that ZNF683 induced NK cell exhaustion motivated us to determine whether ZNF683 also repress effector functions. We first evaluated whether ZNF683 transfection affected expression of cytokines and cytolytic markers. Flow cytometry analyses indicated that IFN‐γ (*P* = .0068) and perforin (*P* = .0011) expression was significantly downregulated in ZNF683 transfection NK cells, whereas CD107a and granzyme B did not differ from that of control NK cells (Figure 6A, Figure S7C,D).

Next, we conducted cytotoxicity assay to directly assess the effect of ZNF683 overexpression on NK cell functionality. Raji cells labelled with luciferase were used as targeted cells, and EV/ZNF683 transfection NK cells were subjected to cytotoxicity assay as effector cells. As shown in Figure 6B, NK cells transfected with EV showed E/T ratio‐dependent cytolytic activity, whereas NK cells with ZNF683 transfection exhibited significantly diminished cytotoxicity at 5:1 and 10:1 E/T ratios, indicating that ZNF683 is the major regulator for NK cells cytotoxicity. In conclusion, we demonstrated that ZNF683 acts as a driving factor in developing exhausted phenotypes, as well as decreasing NK cell cytotoxicity.

### 3.5 Knock down ZNF683 expression in NK cells help reverse NK cell exhaustion in MM patients

To further explore the effect of ZNF683 downregulation on NK cell phenotypes and function in MM patients, we collected NK cells from PB of additional 3 MM patients. We first compared ZNF683 expression in MM‐derived NK cells versus healthy volunteers‐derived NK cells and proved that ZNF683 expression in MM patients is significantly higher than that in healthy volunteers (Figure S8A). ZNF683‐shRNA vector was then used to knock down ZNF683 expression in MM‐derived NK cells. We found that compared with NK cell transfected with empty vectors (EV), exhaustion‐related markers LAG3 (*P* = .0433), CTLA4 (*P* = .0072), TIGIT (*P* = .0223), and CD158b (*P* = .0202) were significantly downregulated (Figure S8B), while activating receptors NKp46 (*P* = .0157) was reversely upregulated in ZNF683 knock down (ZNF683‐KD) NK cells (Figure S8C). IFN‐γ (*P* = .0014) and Perforin (*P* < .0001) were also significant upregulated in ZNF683‐KD NK cells, and Granzyme B had a tendency of upregulation in ZNF683‐KD NK cells versus ZNF683‐EV NK cells (Figure S9A). Meanwhile, cytotoxicity assays showed decreased cytotoxicity in MM‐derived ZNF683^+^ NK cells when compared with healthy volunteers‐derived NK cells, while an increase of NK cell cytotoxicity against target cells was observed in ZNF683‐KD NK cells from MM patients (Figure S9B), further confirming the adverse effect of ZNF683 overexpression on NK cell phenotypes and functions in MM.

### 3.6 SH2D1B is downregulated as a direct target of ZNF683 in NK cells

A progressive downregulation of *SH2D1B* in the pseudo‐temporal trajectory led us to hypothesize that ZNF683 might drive NK cell exhaustion in MM by downregulating *SH2D1B*. Consistent with this idea, *SH2D1B* was expressed at much lower levels in ZNF683^+^ NK cells than other NK cell clusters (Figure 6C). Correlation analysis of *ZNF683* and *SH2D1B* revealed that *SH2D1B* expression exhibited significant negative correlations with *ZNF683* expression (Spearman correlation coefficient r = −.745, *P* = .002) (Figure 6D).

To investigate whether ZNF683 regulates *SH2D1B* expression by directly binding to its promoter region, we predicted binding sites of ZNF683 zinc finger domain on *SH2D1B* by the JASPAR database (http://jaspar.genereg.net/). We identified potential binding sequence (GAGAAAGATAA) among promoter region with relative score of 0.89 on positive‐sense strand, which strongly indicated a direct binding relationship between ZNF683 and *SH2D1B* (Figure 6E). Dual‐luciferase reporter assay was further performed to confirm the prediction results. HEK293 cells containing *SH2D1B* reporter gene with ZNF683 transfection showed significant higher luciferase activity than cells transfected with EV, indicating that transcription factor ZNF683 directly bound to the promoter of *SH2D1B* (Figure 6F). Next, we examined *SH2D1B* expression in NK cells with ZNF683 or EV transfection (*n* = 3). RT‐PCR assays showed that lentiviral transfections of ZNF683 in NK cells markedly abated the expression of *SH2D1B* (Figure 6G). In addition, when we transiently knocked down *SH2D1B*, a significant downregulation of IFN‐γ (*P* = .001) and perforin (*P* = .0222) expression in NK cells were also observed (Figure S9C), indicating that ZNF683‐induced *SH2D1B* downregulation might account for NK cell dysfunction. All these results suggested that ZNF683 significantly induced *SH2D1B* downregulation via directly binding to the promoter of *SH2D1B*.

## 4 DISCUSSION

Crosstalk between malignant plasma cells and their surrounding BM microenvironment plays a pivotal role in MM oncogenesis and progression. Tumour permissive microenvironment‐mediated immune dysfunction is an important mechanism for immune escape of MM cells. Currently, the unprecedented response rates of antibody‐based therapies make investigations on NK cells more and more attractive. Much effort has been devoted to additionally exploit maximization of NK cells‐mediated cytotoxicity in MM. Compared with a great deal of investigations on T cells, studies on the exact mechanism of the interplay between MM cells and NK cells are relatively scarce. Here, we decipher the detailed landscape of NK cells in MM microenvironment and elucidate novel mechanisms for NK cell exhaustion.

Accumulating evidence indicates that cytotoxicity of NK cells are impaired in MM microenvironment.^45^ However, the association between NK cells and the prognosis of MM is still controversial. Jurisic et al.^46^ reported decreased NK cell activity was associated with advanced clinical stage, while Barberi et al.^47^ showed that increased NK cell function was linked to high‐risk disease and ill survival. Moreover, single‐cell RNA‐seq of MM patients‐derived BM samples revealed that NK cell abundance was elevated in asymptomatic stages.^27^ It remains to be confirmed whether the NK cell expansion is a result of MM progression or an indication of antitumour immune response. Due to these controversies, our study conducted single‐cell RNA sequencing and displayed the landscape of NK cell subpopulations and their exact functionalities in MM. We clustered NK cell from MM patients and healthy volunteers into seven distinct subsets and identified a subset of MM‐enriched NK cells. Cluster 3 NK cells exhibited exhaustion phenotypes and damaged cytolytic activities, as well as significant upregulation of ZNF683. NK cells transfected with ZNF683 showed decreased expression of cytolytic molecules, high levels of exhaustion‐related markers, and failed to exert efficient cytotoxicity against tumour cells. Our data indicate that ZNF683 functions as a crucial regulator in promoting NK cell exhaustion. ZNF683 (Hobit) is highly homologous to the transcription factor BLIMP‐1.^48^ Both ZNF683 and BLIMP‐1 have previously been shown to be transcriptional suppressors and induce terminal differentiation in B and T cell lineages. BLIMP‐1 is also well established to upregulate iNKRs and promotes exhaustion of tumour‐infiltrating CD8^+^T cells.^49^ As a homolog of BLIMP‐1, ZNF683 appears to fulfil precondition for a new key transcription factor upregulating inhibitory receptors and immune checkpoints on NK cells. A better understanding of the regulatory relationship between ZNF683 and corresponding receptors may help maximize responsiveness to immunotherapies, such as checkpoint blockade in MM. Remarkably, our research shows that NK cell exhaustion in MM is much more evident in BM than that in PB, implying that NK cells in the suppressive BM microenvironment are more inclined to exhaustion. In addition, our study indicates that there was no difference in the expression of exhaustion‐related molecules before and after treatment, probably due to the short treatment duration. Majority of the patients failed to achieve complete remission after two cycles of VCD regimen. Even CR response might not be enough to reverse NK cell exhaustion. It requires further investigation whether exhaustion could be reversed or not at a prolonged time point (example.g., four or more cycles of treatment) after treatment. Remarkably, failure in reversing NK cell exhaustion by chemotherapy might partly explain why MM is incurable till now. In addition, considering that lenalidomide has been reported to significantly downregulate exhaustion markers in MM‐derived lymphocytes,^50^ VRD regimen might be more efficient in reversing NK cell exhaustion. The choice of using VCD instead of VRD in the current study was for a pragmatic reason (insurance coverage). We are now contemplating future studies to examine whether IMiDs could reverse NK cell exhaustion in MM patients.

EAT‐2 is a vital adaptor in transducing signalling from SLAMF7^17^ for NK cell activation. When the SH2 domain of EAT‐2 binds to Y304 on SLAMF7, PLC‐γ1 and PLC‐γ2 are subsequently recruited to pY127 on EAT‐2, stimulating downstream intracellular calcium mobilization and ERK activation.^51^ The expression of SLAMF7 on both NK and MM cells makes it an ideal target for MM immunotherapy. Elotuzumab (Elo) is a humanized IgG1 mAb targeting SLAMF7, and Elo‐based combination therapies have demonstrated significant clinical activity in relapsed/refractory MM.^52^ It could trigger robust ADCC by NK cells to kill myeloma cells. Collins et al.^53^ demonstrated that except for mediating ADCC, Elo also activated NK cells upon engagement with SLAMF7 and recruiting EAT‐2. However, we found that MM‐derived ZNF683^+^ NK cells hardly expressed EAT‐2 coding gene *SH2D1B*, suggesting that the interaction between SLAMF7 and mAb might fail to trigger activating signalling in ZNF683^+^ NK cells due to EAT‐2 absence. Therefore, the existence of dysfunctional ZNF683^+^ NK cells in MM at least partly explains why a proportion of patients showed ill response to Elo alone or Elo‐based combination therapies.^54^, ^55^ It needs further exploration whether EAT‐2 recovery in ZNF683^+^ NK cells help improve the efficacy of Elo immunotherapies in MM. The analysis of expression dynamics in single‐cell trajectories demonstrated that ZNF683 upregulation and *SH2D1B* downregulation go along with a developmental trajectory towards NK cell exhaustion. We also verified that ZNF683 transfection downregulated *SH2D1B* expression via directly binding to its promoter. Therefore, downregulation of ZNF683 may potentially attenuate and reverse NK cell exhaustion.

In summary, our findings identify a cluster of ZNF683^+^ NK cells with exhaustive phenotypes and impaired cytotoxicity, which contributed to immune escape of MM cells. We further reveal that ZNF683‐induced *SH2D1B* downregulation promotes exhaustion of ZNF683^+^ NK cells and decreases their cytotoxicity, explaining a novel role of NK cell dysfunction in MM oncogenesis. Our research provides new insights into dysregulation of NK cells in MM and offers vital clues for interfering with NK cell exhaustion, so as to develop more effective and more individualized treatments against MM.

## CONFLICT OF INTERESTS

The authors have declared that no conflict of interest exists.

## Supporting information

Supporting Information
