# Supplementary material for: Single‐cell transcriptome profiling reveals the key role of ZNF683 in natural killer cell exhaustion in multiple myeloma
Source: Clin Transl Med. 2022 Oct 17;12(10):e1065. doi: 10.1002/ctm2.1065 (PMC9574488; doi:10.1002/ctm2.1065)
Supplement: Supplementary file 1 — Supporting Information [file CTM2-12-e1065-s001.docx]

**Single-cell transcriptome profiling reveals the key role of ZNF683 in NK cell exhaustion in multiple myeloma**

**Supporting Information**

1. **Supplementary figures & legends 1-9**
2. **Supplementary tables 1-2**

**Supplementary figures**

**
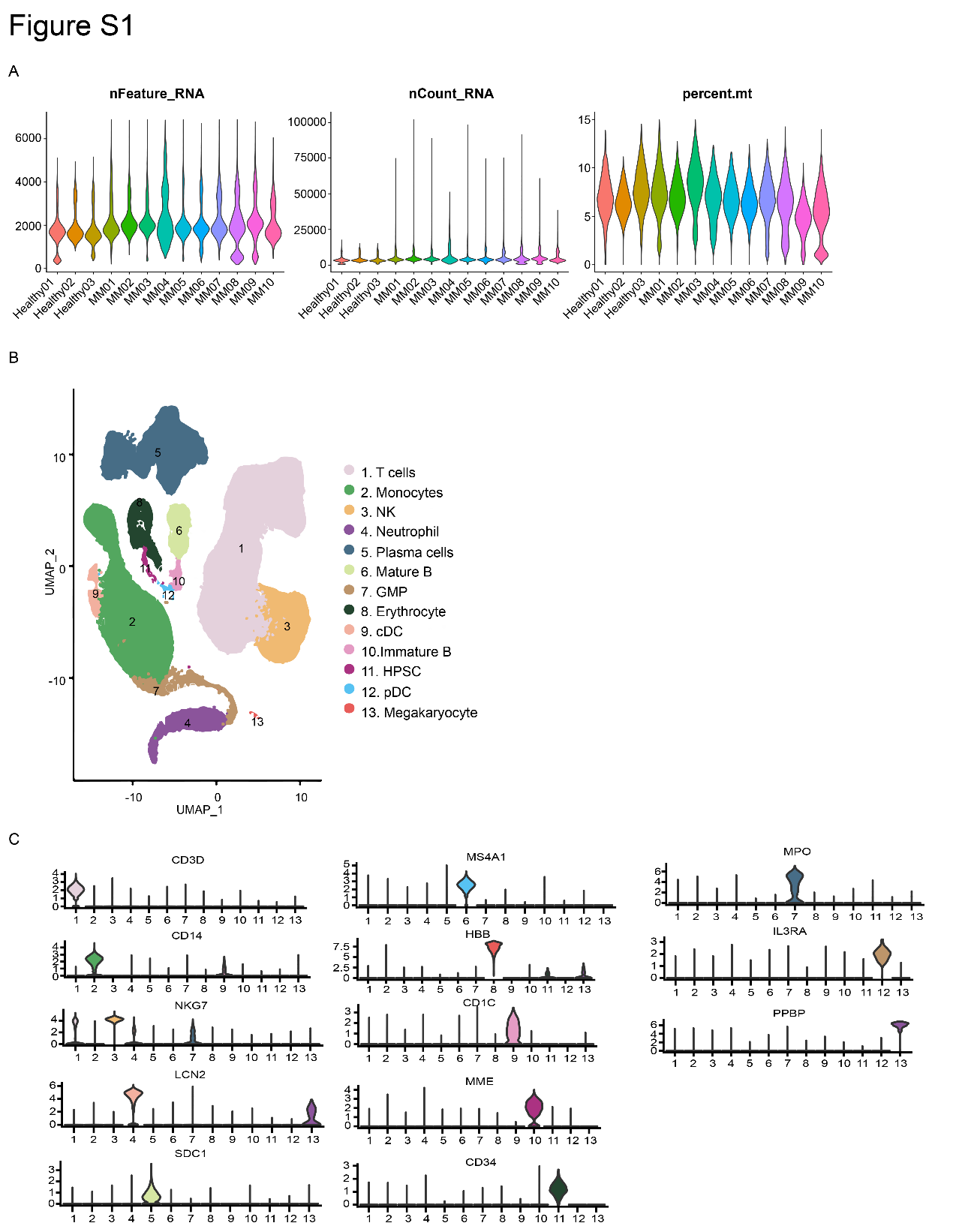
**

**Supplementary Figure 1. The cellular landscape in healthy and MM samples.** (**A**) QC plots after filtering. (**B**) UMAP plot showing 241440 single cells from MM patients and healthy volunteers-derived BM/ PB samples grouped into diverse cell types. All included cells were color-coded according to the normalized expression levels of marker genes and were identified as T cells, NK cells, monocytes, mature B cells, cDC, pDC, megakaryocyte, plasma cells, immature B cells, hPSC, Erythrocyte, GMP, neutrophils. Each dot in the UMAP plot represented a single cell. (**C**) Violin plots exhibited the expression of marker gene(s) for each cluster. Cell clusters and the expressions were indicated at the x- and y-axis, respectively.

**
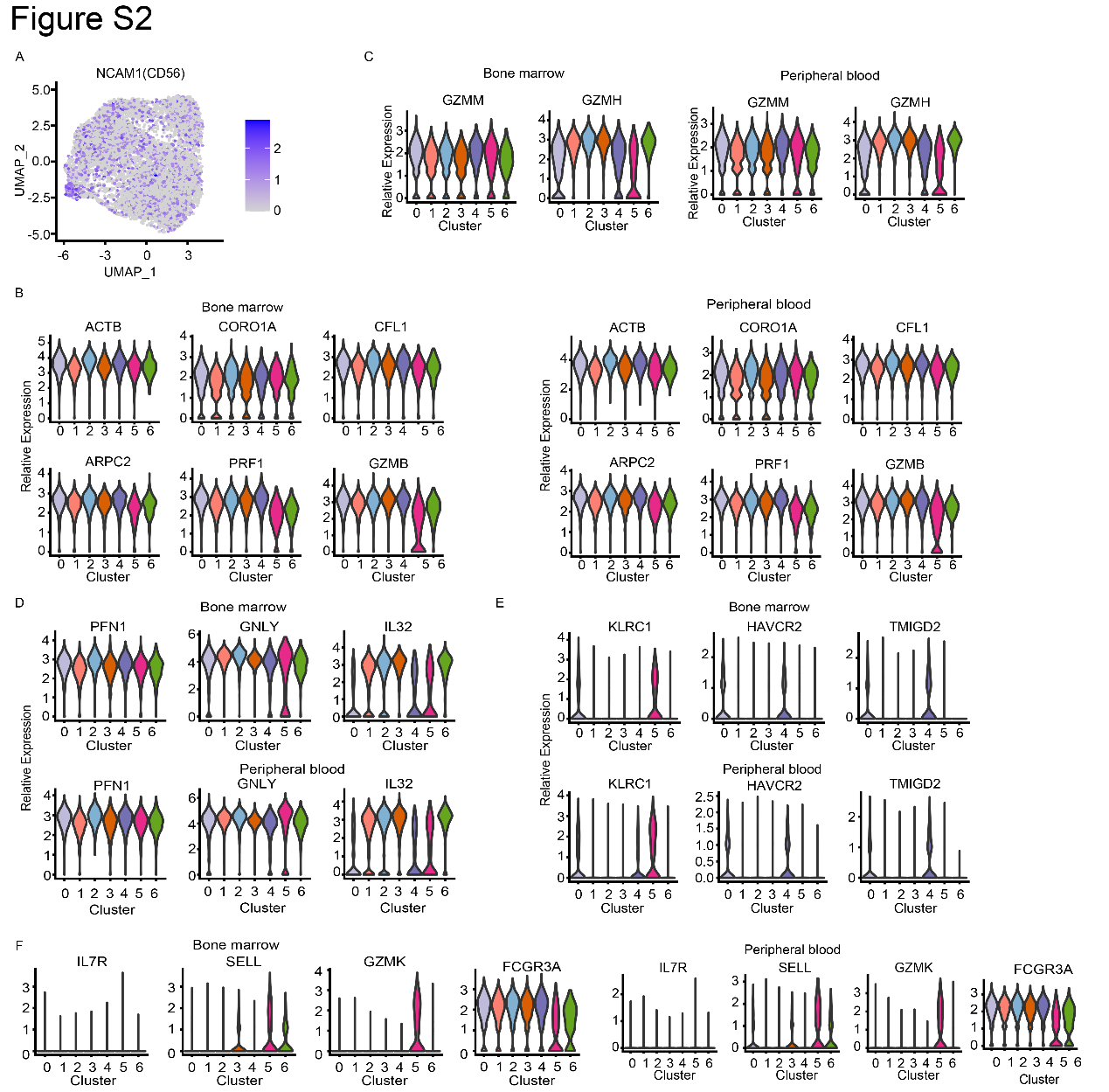
**

**Supplementary Figure 2. Expression profiles of different clusters of NK cells.** (**A**) Feature plots of *NCAM1* expression in each NK cluster. (**B**) Representative genes associated with CD56^dim^ effector NK cells were plotted via violin plots of BM and PB samples. (**C**) Expression of cytotoxicity-related genes GZMM and GZMH in each NK cluster. (**D**) Violin plots showing genes highly expressed in active NK cells. (**E**) Violin plots indicating maturity-related genes in each NK cluster. (**F**) Violin plots showing the expression of genes known to be highly expressed in CD56^bright^ NK cells.


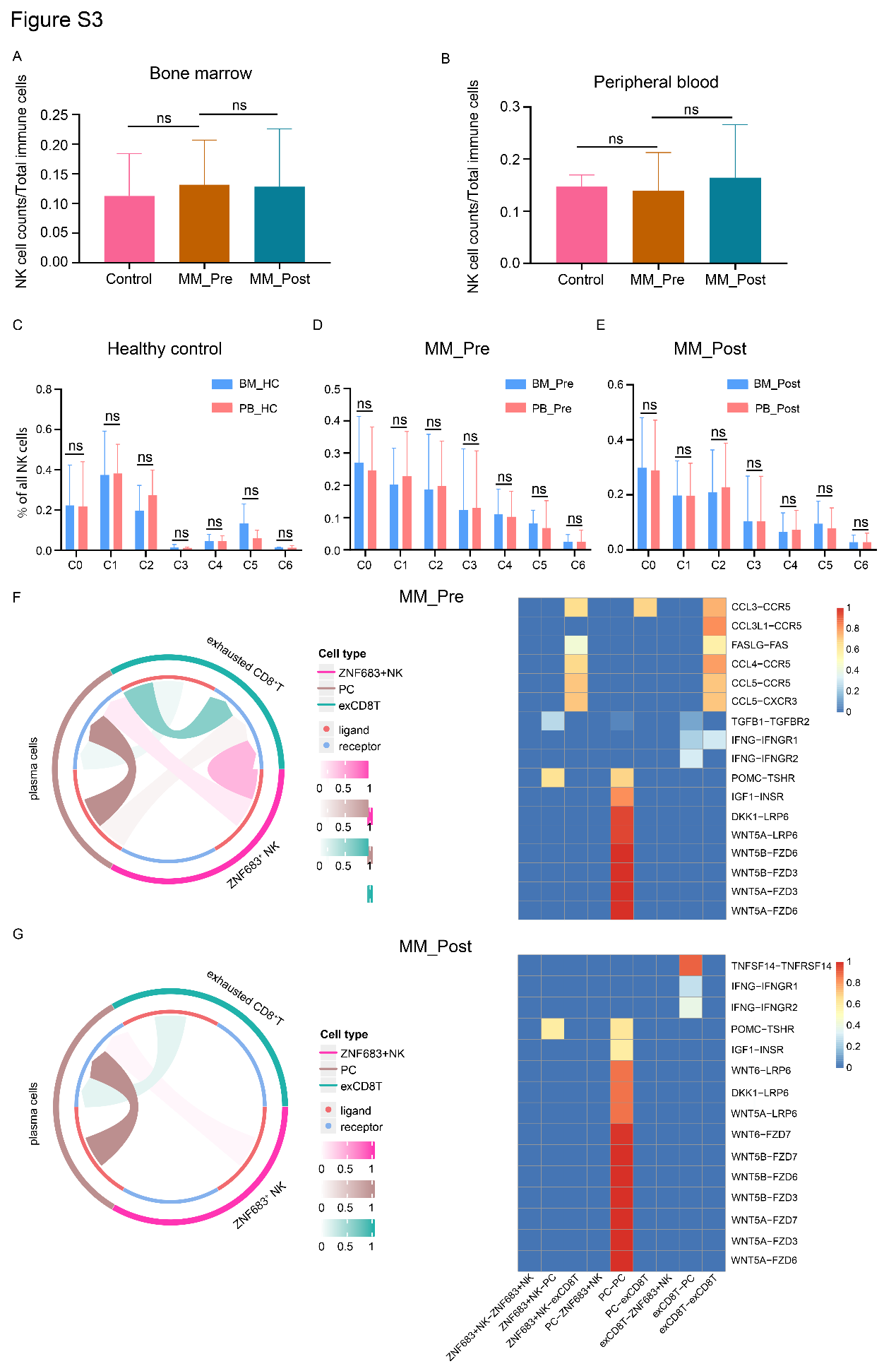


**Supplementary Figure 3. Proportional differences of NK cells between MM patients and healthy volunteers. (A-B)** Proportion of NK cells in BM aspirates (A) and PB (B) of healthy volunteers (control), MM patients before treatment (MM_Pre), and MM patients after treatment(MM_Post). (**C-E**) The proportion of each NK cluster in total NK cells between BM and PB in healthy volunteers (C), MM patients before (D) and after treatment (E). (**F-G**) The intercellular communication signals among ZNF683+ NK cells, exhausted T cells and plasma cells of MM patients before (F) and after treatment (G) by Cellcall. ns, not significant.

**
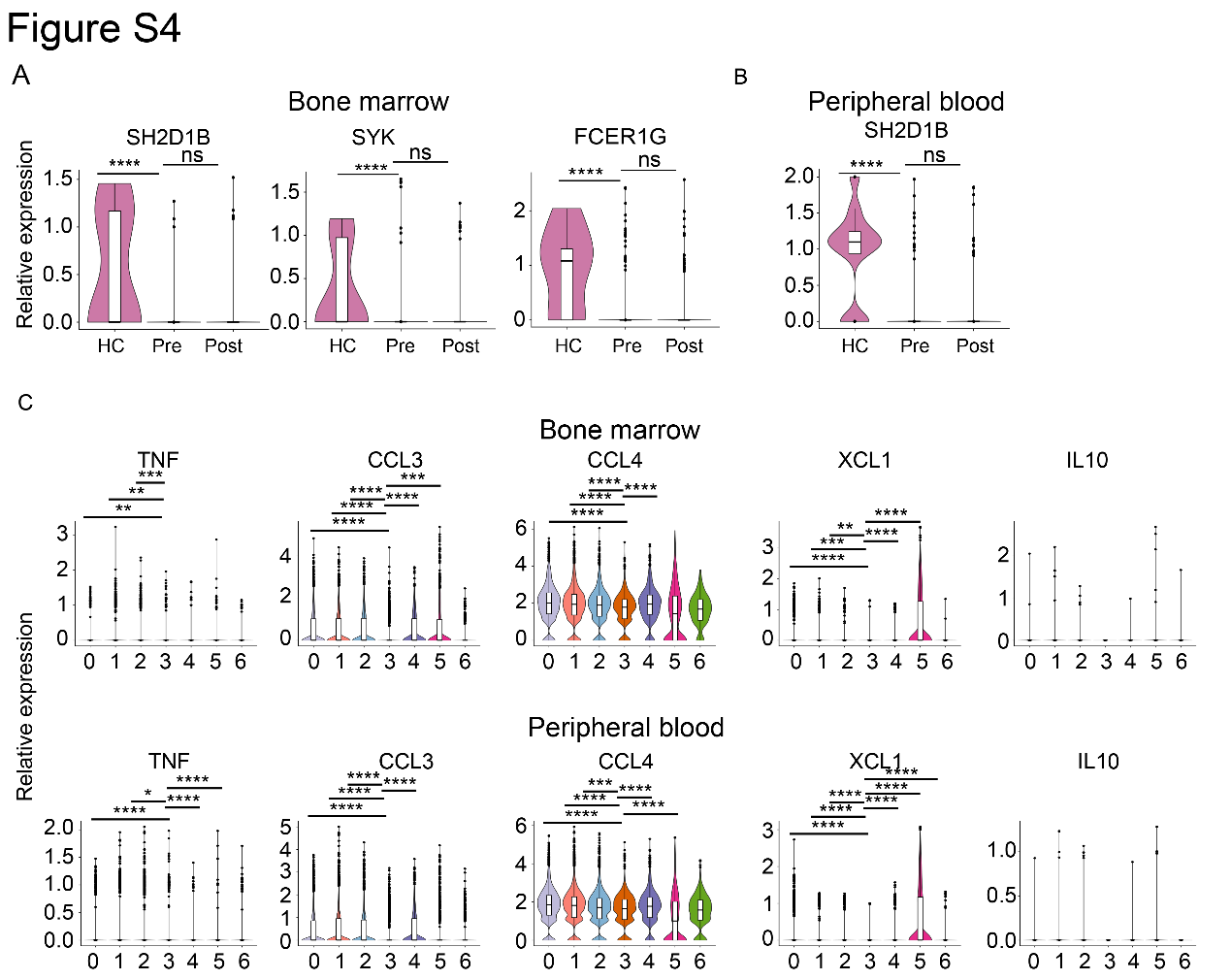
**

**Supplementary Figure 4. Expression of cytotoxicity/apoptosis-related genes** **in ZNF683^+^ NK cells.** (**A** and **B**) Violin plot depicting expression of NK cell cytotoxicity-related genes in ZNF683^+^ NK cells from healthy volunteers and MM patients. Selected genes from BM (A) and PB (B) were extracted from KEGG results in Figure 2A. (**C**) Violin plots compare mRNA expression of cytokines in all NK clusters. **P* < 0.05, ***P* < 0.01, ****P*< 0.001*****P*< 0.0001, by Wilcoxon rank-sum test.

**
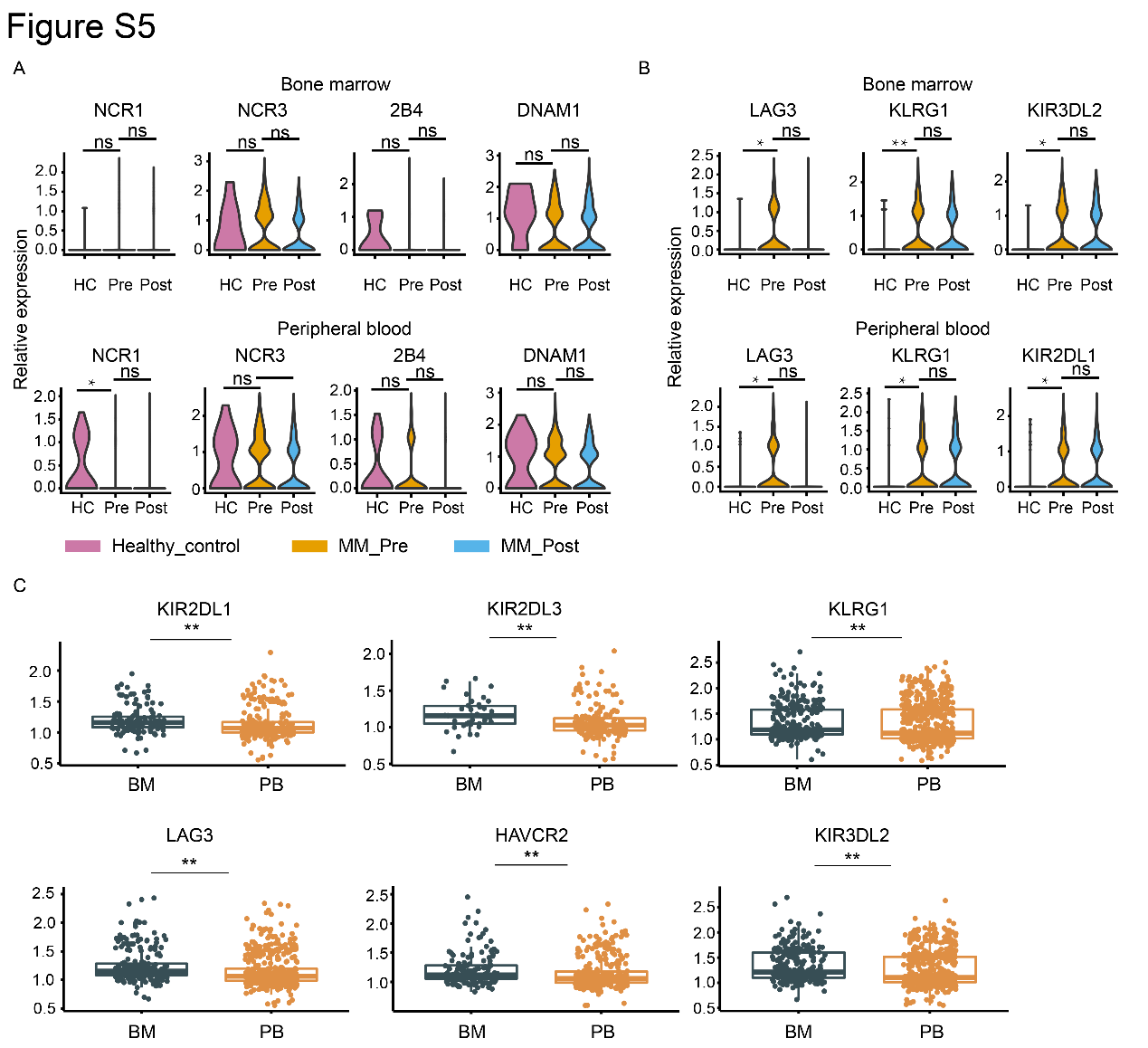
**

**Supplementary Figure 5. Expression of activating/inhibitory receptors on ZNF683^+^ NK cells.** (**A**) Violin plots depict expression of marker genes for activating receptors in BM/PB ZNF683^+^ NK cells from healthy volunteers and MM patients. (**B**) Violin plots show expression of marker genes for inhibitory receptors in BM/PB ZNF683^+^ NK cells from healthy volunteers and MM patients. (**C**) Box plots compare expression of marker genes for inhibitory receptors on MM patients-derived ZNF683^+^ NK cells between BM and PB. **P*< 0.05, ** *P*< 0.01, by Wilcoxon rank-sum test.

**
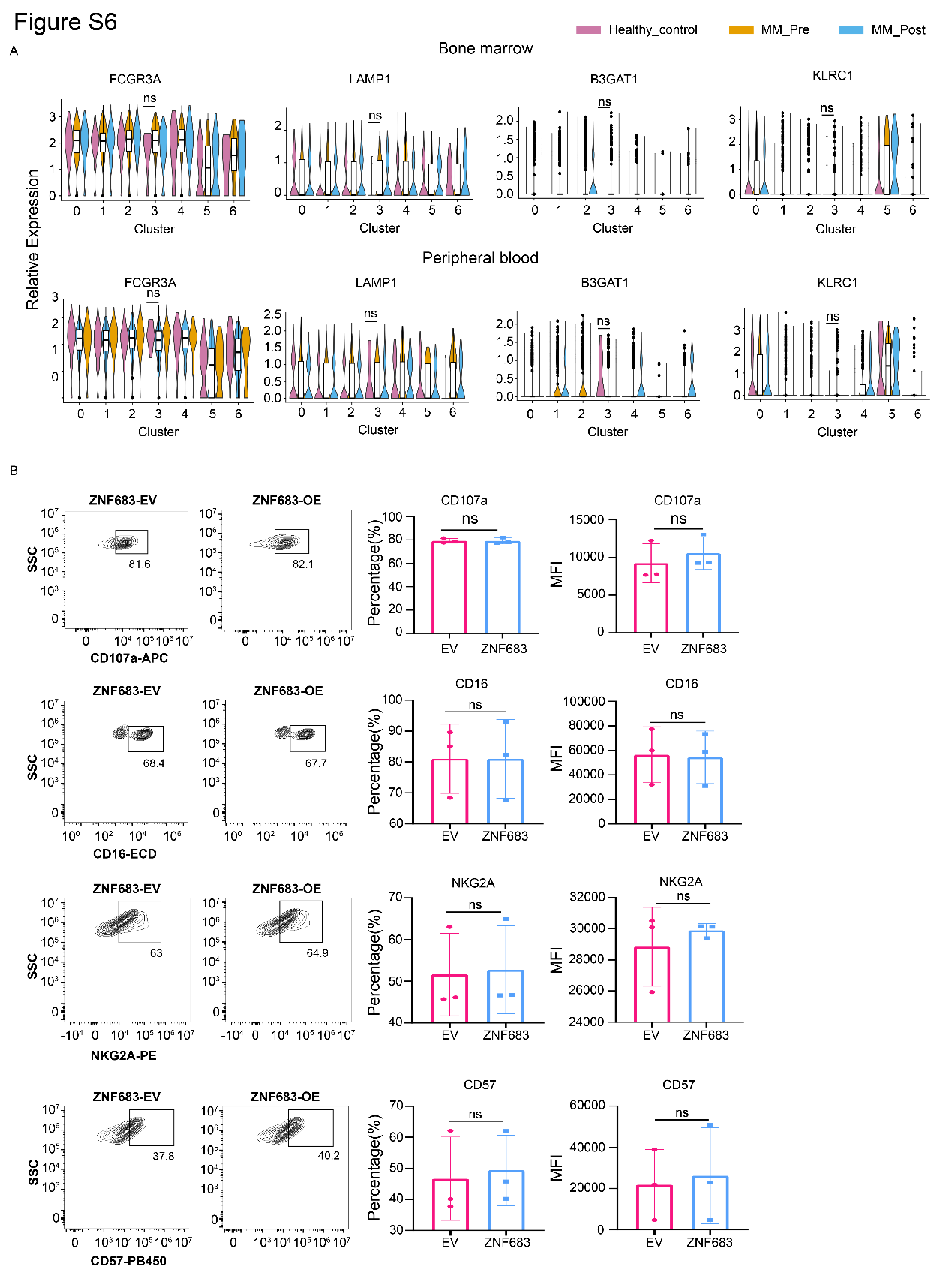
**

**Supplementary Figure 6. Anergy and senescence phenotype related gene expression on NK cells.** (**A**) The expression profile of anergy related gene (*FCGR3A*, *LAMP1*) and senescence related gene (*B3GAT1*, *KLRC1*) in BM and PB of MM patients and healthy volunteers. (**B**) The protein expression of anergy markers (CD16, CD107a) and senescence markers (CD57, NKG2A) in BM and PB of MM patients and healthy volunteers.

**
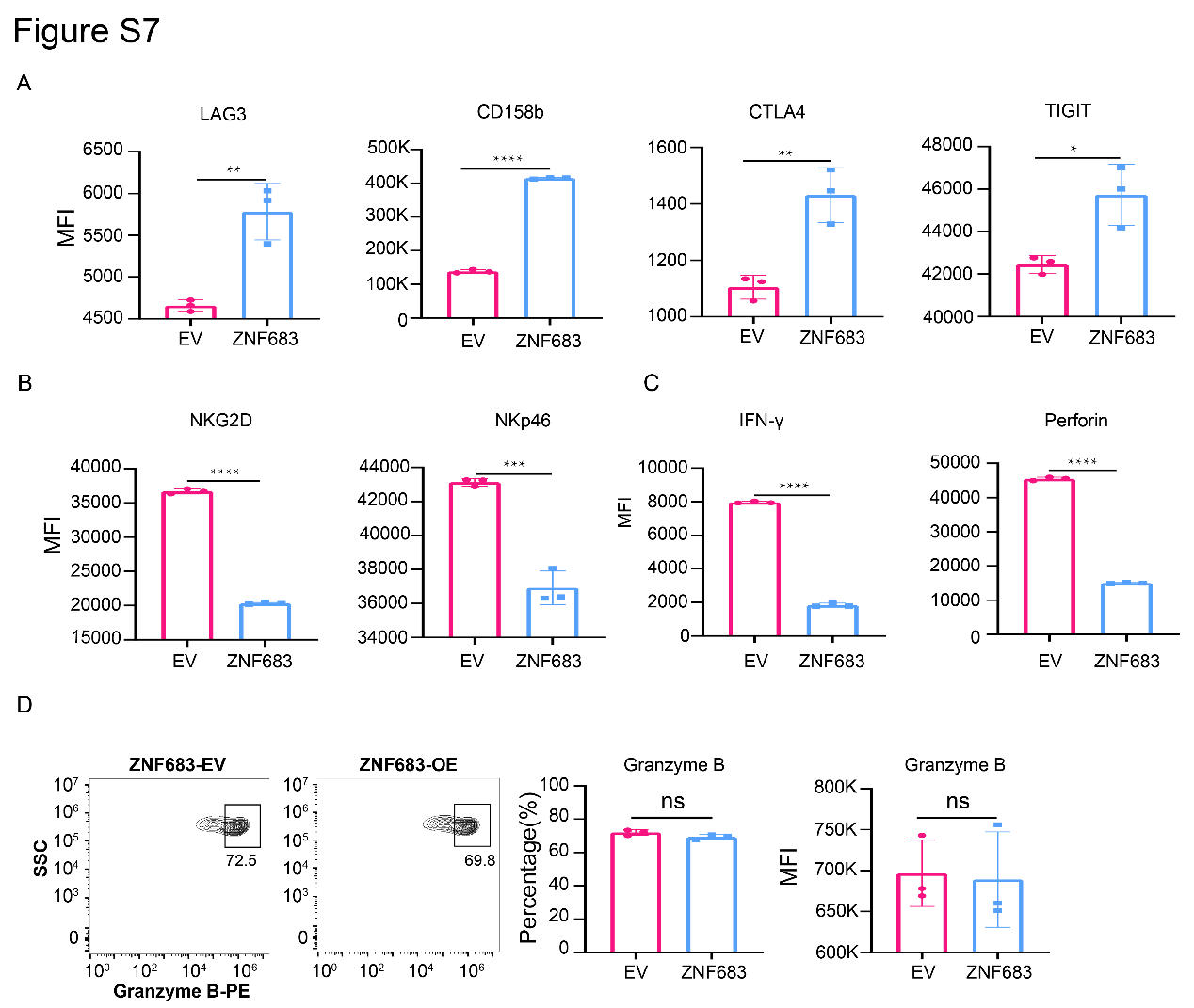
**

**Supplementary Figure 7. The effect of ZNF683 overexpression on Mean Fluorescence Intensity (MFI) for the receptors.** (**A**) NK cells were isolated from PB of healthy volunteers (n=3), then they were transfected with ZNF683 overexpressing vectors (ZNF683) or empty vectors (EV). Flow cytometry result demonstrates the effect of ZNF683 transfection on the Mean Fluorescence Intensity (MFI) for the inhibitory receptors LAG3, CD158b, CTLA4, and TIGIT. (**B**) The MFI for the activating receptors NKG2D and NKp46. (**C**) The MFI for the cytokine IFN-γ and cytotoxic perforin. (**D**) NK cells were isolated from PB of healthy volunteers (n=3), then they were transfected with ZNF683 overexpressing vectors (ZNF683) or empty vectors (EV). Flow cytometry result demonstrates the effect of ZNF683 transfection on expression (percentage/MFI) of Granzyme B. **P* < 0.05; ***P*< 0.01; ****P*< 0.001; *****P*< 0.0001; ns, not significant, by two-tailed Student’s t test.


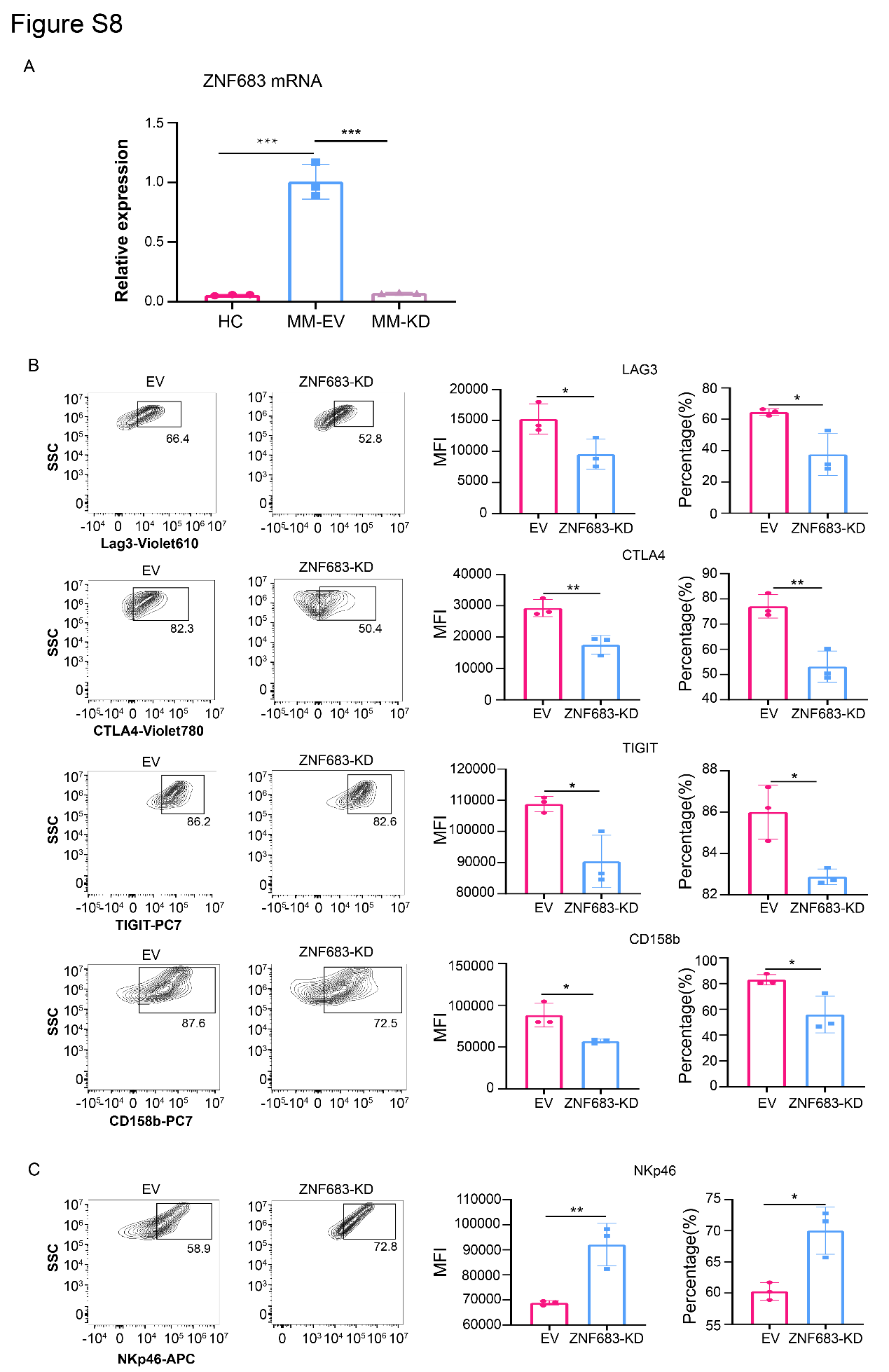


**Supplementary Figure 8. The effect of ZNF683 knockout on MM-derived NK cell phenotypes.** (**A**) NK cells isolated from PB of MM patients (n=3) were transfected with ZNF683-shRNA vectors (ZNF683-KD) or EV. RT-qPCR revealed ZNF683 expression in healthy controls-derived NK cells (HC), MM patients-derived NK cells with EV transfection (MM-EV), and MM patients-derived NK cells with ZNF683 knockout (MM-KD). (**B**) Flow cytometry result demonstrates the effect of ZNF683 knockout on the expression of inhibitory receptors LAG3, CD158b, CTLA4, and TIGIT. (**C**) Flow cytometry result demonstrates the effect of ZNF683 knockout on the expression of activating receptor NKp46. **P* < 0.05; ***P*< 0.01; *** *P*< 0.001, by two-tailed Student’s t test.


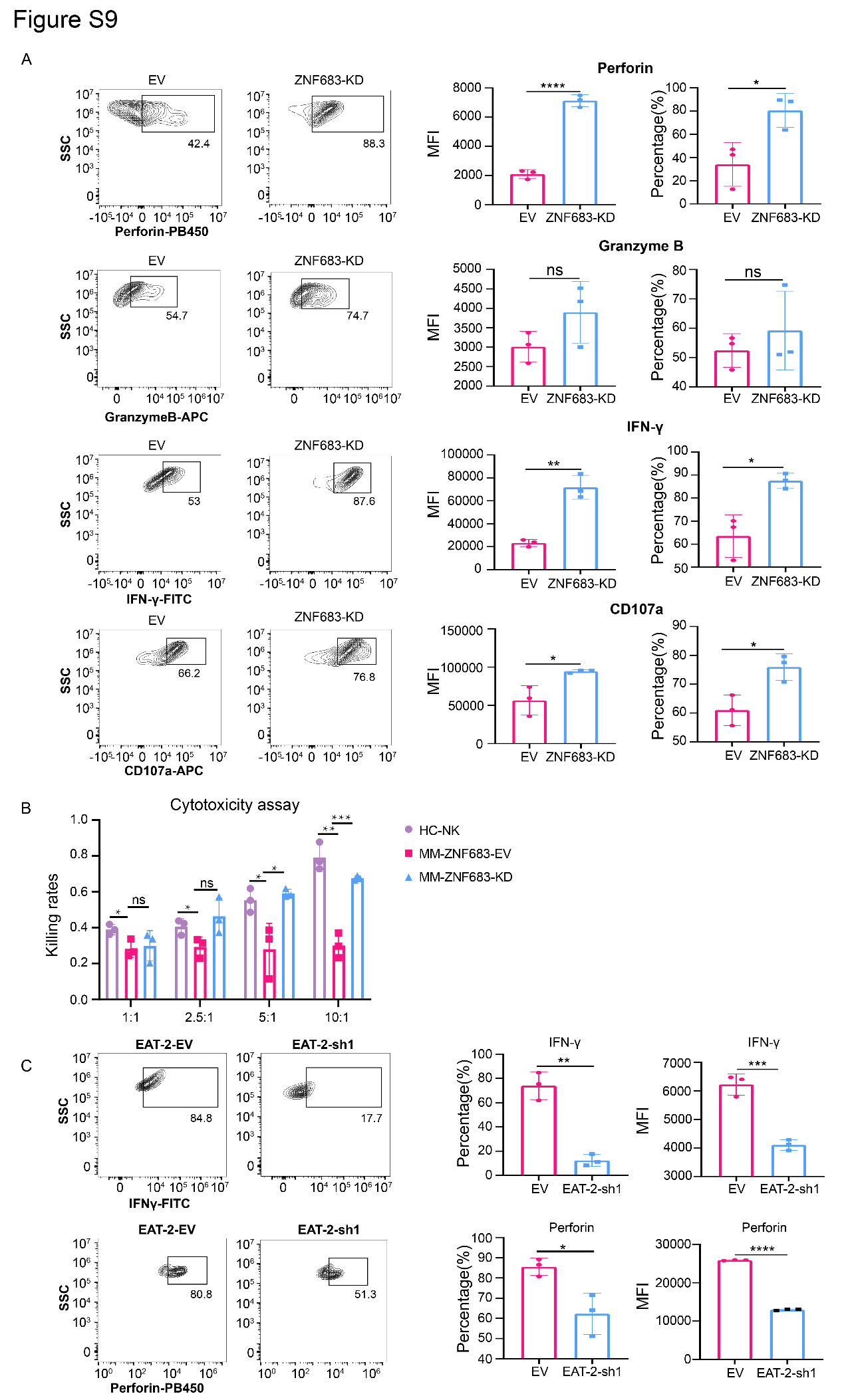


**Supplementary Figure 9. The effect of ZNF683/SH2D1B knockout on NK cell function.** (**A**) NK cells isolated from PB of MM patients (n=3) were transfected with ZNF683-shRNA vectors (ZNF683-KD) or EV. Flow cytometry result demonstrates the effect of ZNF683 knockout on the expression of cytolytic Granzyme B and perforin, as well as IFN-γ and CD107a. (**B**) Cytotoxicity assay illustrated the effect of ZNF683 transfection on NK cell cytotoxicity (n=3 per group). (**C**) NK cells isolated from PB of healthy volunteers (n=3) were transfected with *SH2D1B* shRNA (EAT-2-sh1) or EV. Flow cytometry assay show effect of SH2D1B downregulation on IFN-γ and perforin expression (percentage/MFI) in NK cells. **P* < 0.05; ***P*< 0.01; ****P*< 0.001; *****P*< 0.0001; ns, not significant, by two-tailed Student’s t test.

**Supplementary tables**

**Supplementary Table 1. Oligonucleotides for shRNA**

| Oligo-nucleotides | Sequences (5’to 3’) |
| --- | --- |
| Primer-NC-T | GATCTGTTCTCCGAACGTGTCACGTTTCAAGAG  AACGTGACACGTTCGGAGAATTTTTTC |
| Primer-NC-B | AATTGAAAAAATTCTCCGAACGTGTCACGTTCT  CTTGAAACGTGACACGTTCGGAGAACA |
| Primer-T1-SH2D1B | GATCCGCGATTATGTGGATGTCTTGCCTCGAGG  CAAGACATCCACATAATCGCTTTTTT |
| Primer-B1-SH2D1B | AATTAAAAAAGCGATTATGTGGATGTCTTGCCT  CGAGGCAAGACATCCACATAATCGCG |
| Primer-T2-SH2D1B | GATCCGCAAGACTGTGAGACCTTGCTCTCGAGA  GCAAGGTCTCACAGTCTTGCTTTTTT |
| Primer-B2-SH2D1B | AATTAAAAAAGCAAGACTGTGAGACCTTGCTCT  CGAGAGCAAGGTCTCACAGTCTTGCG |
| Primer-T3-SH2D1B | GATCCGAGGATTGAAATTAGAGTTGGCTCGAGC  CAACTCTAATTTCAATCCTCTTTTTT |
| Primer-B3-SH2D1B | AATTAAAAAAGAGGATTGAAATTAGAGTTGGCT  CGAGCCAACTCTAATTTCAATCCTCG |
| Primer-T1-ZNF683 | GATCCGCTGCACAATTAGGTTGTTGTCTCGAGAC  AACAACCTAATTGTGCAGCTTTTTT |
| Primer-B1-ZNF683 | AATTAAAAAAGCTGCACAATTAGGTTGTTGTCTCG  AGACAACAACCTAATTGTGCAGCG |
| Primer-T2-ZNF683 | GATCCGCTCCACCGATGACAAGAAATCTCGAGATT  TCTTGTCATCGGTGGAGCTTTTTT |
| Primer-B2-ZNF683 | AATTAAAAAAGCTCCACCGATGACAAGAAATCTCG  AGATTTCTTGTCATCGGTGGAGCG |
| Primer-T3-ZNF683 | GATCCGCCTGCTGATGATGGTCAATGCTCGAGCATT  GACCATCATCAGCAGGCTTTTTT |
| Primer-B3-ZNF683 | AATTAAAAAAGCCTGCTGATGATGGTCAATGCTCG  AGCATTGACCATCATCAGCAGGCG |

**Supplementary Table 2. Sequences of RT-qPCR primers**

|  | Sequence (5’-3’) |
| --- | --- |
| ZNF683-F | GAGAGCGTCCATTCCAGTGT |
| ZNF683-R | CAGGTGGGTCTTGAGGTTA |
| SH2D1B-F | CGAATCTTCAGAGAGAAACACG |
| SH2D1B-R | GGGCTGGTTCTCTTTATTGG |
| GAPDH-F | GCCAAAAGGGTCATCATCTC |
| GAPDH-R | TGTGGTCATGAGTCCTTCCA |
